# Arcuate hypothalamic AgRP and putative POMC neurons show opposite changes in spiking across multiple timescales

**DOI:** 10.7554/eLife.07122

**Published:** 2015-07-10

**Authors:** Yael Mandelblat-Cerf, Rohan N Ramesh, Christian R Burgess, Paola Patella, Zongfang Yang, Bradford B Lowell, Mark L Andermann

**Affiliations:** 1 Department of Endocrinology, Beth Israel Deaconess Medical Center, Boston, United States; 2 Program in Neuroscience, Harvard Medical School, Boston, United States; University of California, San Diego, United States

**Keywords:** AgRP, tetrode, POMC, feeding, food cue, arcuate, mouse

## Abstract

Agouti-related-peptide (AgRP) neurons—interoceptive neurons in the arcuate nucleus of the hypothalamus (ARC)—are both necessary and sufficient for driving feeding behavior. To better understand the functional roles of AgRP neurons, we performed optetrode electrophysiological recordings from AgRP neurons in awake, behaving *AgRP-IRES-Cre* mice. In free-feeding mice, we observed a fivefold increase in AgRP neuron firing with mounting caloric deficit in afternoon vs morning recordings. In food-restricted mice, as food became available, AgRP neuron firing dropped, yet remained elevated as compared to firing in sated mice. The rapid drop in spiking activity of AgRP neurons at meal onset may reflect a termination of the drive to find food, while residual, persistent spiking may reflect a sustained drive to consume food. Moreover, nearby neurons inhibited by AgRP neuron photostimulation, likely including satiety-promoting pro-opiomelanocortin (POMC) neurons, demonstrated opposite changes in spiking. Finally, firing of ARC neurons was also rapidly modulated within seconds of individual licks for liquid food. These findings suggest novel roles for antagonistic AgRP and POMC neurons in the regulation of feeding behaviors across multiple timescales.

**DOI:** http://dx.doi.org/10.7554/eLife.07122.001

## eLife digest

Appetite is controlled in part by the opposing actions of the ‘hunger hormone’ (called ghrelin) and the ‘satiety hormone’ (called leptin). Ghrelin is released by the stomach when empty and stimulates appetite, whereas leptin is released by fat stores and induces feelings of fullness. Both hormones travel via the bloodstream and are detected by a region of the brain called the hypothalamus.

Ghrelin and leptin act specifically on a group of cells in the hypothalamus that contains at least two major cell types: AgRP neurons and POMC neurons. Electrode recordings from slices of mouse brain show that AgRP neurons fire more rapidly at night—when mice normally feed—than during the day, whereas POMC neurons do the opposite. This suggests that the activity of AgRP neurons drives food-seeking behavior, whereas POMC firing inhibits it. However, the absence of circulating hormones such as leptin and ghrelin in brain slices makes it difficult to draw firm conclusions about the role of these cells in controlling appetite.

Mandelblat-Cerf, Ramesh, Burgess et al. have addressed this issue by performing the first recordings of spiking activity in individual AgRP neurons and other cells that are likely to be POMC neurons in awake mice. Consistent with the results of slice experiments, the firing rate of AgRP neurons increased steadily over the course of the day, suggesting that their activity signals an increasing need for food. Furthermore, as soon as food became available, the firing rate of the AgRP neurons suddenly dropped—even though the animals' energy reserves would still have been low. These results are consistent with the findings of two recent studies reported earlier this year that used different methods to indirectly measure neuronal activity in awake mice. Notably, even after the drop in activity, the firing rates of AgRP neurons remained above those recorded in fully sated mice—which possibly reflects the fact that the animals' energy reserves were still low. The putative POMC neurons generally showed opposite effects to the AgRP neurons.

The results of these electrode recordings in awake mice thus suggest that AgRP and POMC neurons together maintain a drive to seek out food sources as energy reserves fall, and to refrain from doing so when energy reserves are plentiful. Moreover, the seemingly paradoxical drop in AgRP firing and increase in POMC firing upon receiving food may act as a signal to temporarily stop searching for food, so that feeding itself can begin. Alternatively, since the release of satiety hormones after eating a meal is slow, these rapid changes in firing may provide more immediate feedback to the neuronal circuits that regulate the drives to seek and consume food.

**DOI:** http://dx.doi.org/10.7554/eLife.07122.002

## Introduction

The homeostatic drive to feed is at least partially driven by agouti-related-peptide (AgRP) neurons in the arcuate nucleus of the hypothalamus (ARC). These neurons have privileged access to slow hormonal signals of energy balance, such as ghrelin and leptin (Willesen et al., 1999, Morton and Schwartz, 2001, Zigman and Elmquist, 2003, Varela and Horvath, 2012, Wang et al., 2014), and also receive long-range glutamatergic, GABAergic, and peptidergic synaptic inputs from multiple central brain nuclei, including the paraventricular and dorsomedial hypothalamus (Krashes et al., 2014). Both opto- and pharmaco-genetic activation of AgRP neurons drive intense feeding in ad libitum-fed mice (Aponte et al., 2011, Krashes et al., 2011), while loss-of-function experiments in food-restricted mice lead to a reduction in food consumption (Gropp et al., 2005, Luquet et al., 2005, Krashes et al., 2011). These studies suggest that AgRP neurons represent a critical node in the neural pathway (or pathways) linking interoceptive sensing of energy deficit with the decision to seek and consume food. A requirement for pinpointing the precise role of AgRP neurons in driving various aspects of this complex feeding process involves the direct evaluation of their endogenous spiking activity.

Previous attempts to directly record spiking activity of AgRP neurons have been restricted to in vitro approaches, due to the technical challenges of extracellular electrophysiological recordings in the ARC in living animals, and the fact that AgRP neurons are intermingled with pro-opiomelanocortin (POMC) neurons with opposing effects on food intake (Varela and Horvath, 2012, Zhan et al., 2013). Consistent with the hypothesized enhancement of AgRP neuron firing in times of caloric deficit, in vitro recordings of AgRP neurons in brain slices from mice during their dark cycle or during a period of fasting revealed enhanced action potential firing and spontaneous subthreshold currents as compared to recordings during the light cycle (Yang et al., 2011, Liu et al., 2012, Krashes et al., 2013). Interestingly, opposite effects were observed in satiety-promoting POMC neurons (Yaswen et al., 1999, Aponte et al., 2011, Zhan et al., 2013), which are known to be inhibited by GABA release from AgRP neurons (Cowley et al., 2001, Atasoy et al., 2012). Studies performed in vitro further suggest that AgRP and POMC neurons exert antagonistic influences, not only on each other's activity (Cone et al., 2001, Yang et al., 2011, Atasoy et al., 2012), but also on the activity of common long-range target nuclei (Bagnol et al., 1999, Cowley et al., 1999, Atasoy et al., 2012, Atasoy et al., 2014). However, these in vitro experiments were performed under conditions in which most endogenous circulating factors are absent, and most sources of slow and fast afferent neuronal input are severed. Indeed, in the presence of realistic levels of synaptic inhibition, recordings from de-afferented AgRP neurons show minimal action potential firing in vitro (Yang et al., 2011). High-temporal resolution in vivo recordings of spiking activity in identified single neurons in the intact ARC would be necessary to confirm these findings regarding sensitivity to slow changes in energy deficit and could potentially reveal novel roles for AgRP and other ARC neurons in guiding food-seeking and feeding behaviors at shorter timescales.

Here, we used an optetrode approach to investigate the in vivo spiking activity of AgRP neurons and a group of nearby neurons inhibited by AgRP neuron photostimulation (ARC_inh_ neurons). Because the only ARC neurons currently known to be inhibited by AgRP neurons are POMC neurons (Cowley et al., 2001, Atasoy et al., 2012), a large fraction of ARC_inh_ neurons are likely to be POMC neurons. We found that AgRP neuron firing rates increased across hours over the course of the light period. Surprisingly, we also found that AgRP neurons exhibited a sudden and sustained decrease in spiking over the course of minutes, in response to feeding as well as to cues that predicted the availability of food. This decreased level of spiking, which persisted throughout the meal, nevertheless exceeded spiking rates during recordings from ad libitum fed, sated mice at the onset of the light cycle. This abrupt change in spiking cannot simply reflect homeostatic changes but instead suggests that the drop in AgRP neuron spiking reflects a reduction in the drive to seek food at the initiation of food consumption. Finally, we observed that the activity of ARC neurons could be modulated on the timescale of seconds by feeding-related behaviors, including individual licks for liquid food. In general, neurons inhibited by AgRP neuron photostimulation show opposite effects to AgRP neurons. Together, these results suggest that, in addition to sensing slow systemic changes in energy balance, AgRP and POMC neurons may also integrate this information with complex environmental cues regarding food availability and feeding context, in order to dynamically adjust feeding behaviors at timescales from hours to seconds.

## Results

### Extracellular recordings of ARC neurons in awake mice

**Figure 1.**
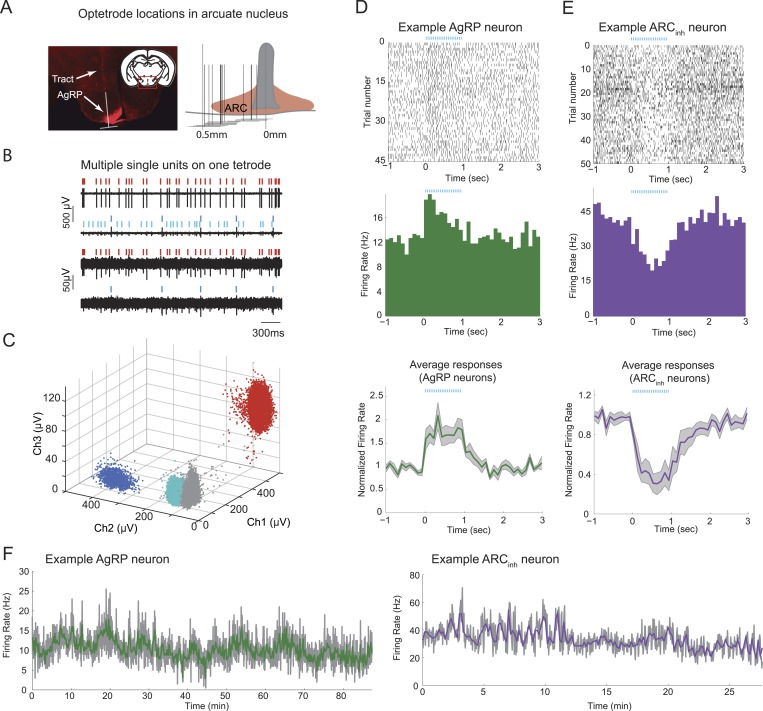
Stable optetrode recordings from arcuate hypothalamic neurons. (**A**) An optetrode was implanted into the arcuate nucleus of the hypothalamus to identify genetically-defined, ChR2-mCherry-expressing agouti-related-peptide (AgRP) neurons (see below and ‘Materials and methods’). Left: coronal section, 1.5 mm posterior to Bregma (inset) and example histological section, showing AgRP neurons in the ARC (mCherry expression, red), and localization of optetrode recording site (as determined by visualization of optetrode track). White inverted ‘T’ shape denotes location of optetrode track (vertical line) and approximate width of optetrode (horizontal line), which estimates the medial-lateral range of potential locations of recorded single-units. Right: schematic showing optetrode locations across 12 mice for which optetrode tracks were recovered. (**B**) Example voltage traces from recordings of spontaneous spiking from one tetrode. Note differences in scale bar across electrode channels, reflecting difference in waveform amplitude across channels. (**C**) Cluster-plots showing discriminability of spikes from different cells using tetrodes. Each dot represents the peak amplitude of a single-spike waveform, measured on three different electrodes within the four-wire tetrode bundle. In this example, each spike waveform was designated as belonging to one of three separable single-units (colored dots), or to multi-unit activity (gray dots). Colors for different single-units match the ticks above the spike traces in **B**. (**D**) Example of a single-unit defined as a putative AgRP neuron, with peri-photostimulation (blue lines) spike raster plot (top), average peri-stimulus time histogram (PSTH) across trials (middle), and mean normalized PSTH (average of individual neuron PSTHs after normalization by pre-pulse-train firing rate) across all 19 AgRP neurons recorded from 9 ad libitum-fed mice (bottom). Shaded areas denote SEM. (**E**) Raster and PSTH plots (top, middle) for an example single-unit defined as significantly and strongly (>20%) inhibited by AgRP neuron photostimulation (ARC_inh_), and mean normalized PSTH (bottom) across all ARC_inh_ units in ad libitum-fed mice (n = 14). (**F**) Firing rate timecourses, in 2-s bins (gray) and 10-s bins (colored), for the two example cells in **D** and **E**. In ad libitum-fed mice in the absence of food cues or food, AgRP neurons and ARC_inh_ neurons exhibited stable minute-to-minute firing rates across recordings ranging from 30 to 90 min. **DOI:** http://dx.doi.org/10.7554/eLife.07122.003

We recorded spiking activity from AgRP neurons and other nearby ARC neurons, by selectively expressing cre-dependent channelrhodopsin (AAV9-FLEX-hSYN-ChR2-mCherry) in the ARC of *Agrp-IRES-Cre* mice, and by subsequently performing extracellular optetrode recordings in awake, behaving mice in which putative AgRP neurons were identified via a significant increase in firing during optogenetic photostimulation (see below). Because AgRP neurons are densely packed in the ARC (Figure 1A), we used tetrodes with high impedance that allowed for isolation of large spike waveforms (Figure 1B) from neurons proximal to the tetrodes (4–8 bundles of 4 wires, <70 µm total diameter per bundle). Several neurons could be recorded and discriminated on each tetrode and clustered via differences in spike waveform amplitudes across electrodes within a tetrode (Figure 1B,C). We verified that recordings were located within the ARC; Figure 1A.

**Figure 1—figure supplement 1.**
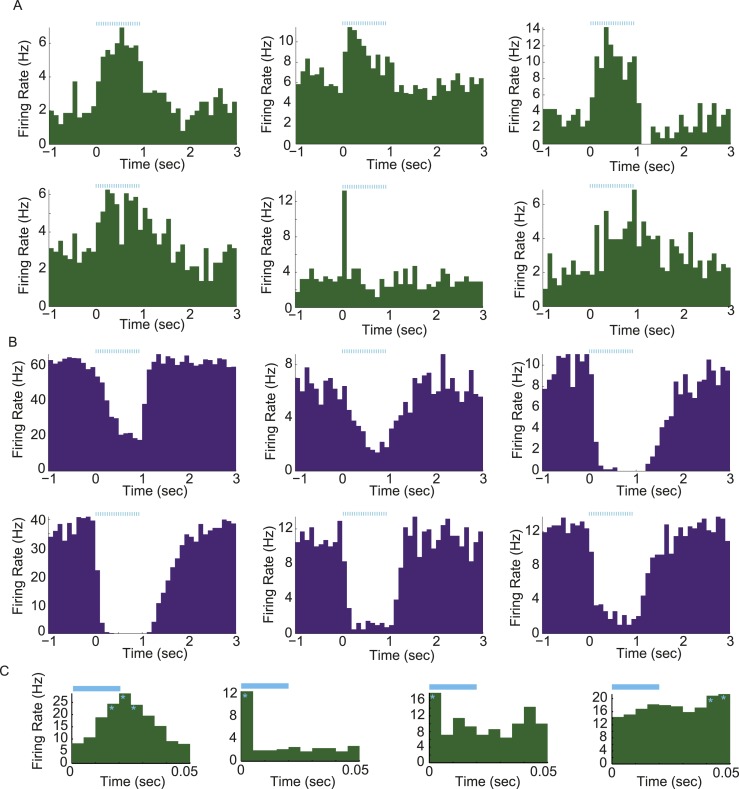
Light-evoked responses in different populations of arcuate neurons. (**A** and **B**) Activity aligned to the onset of a train of photostimulation (blue ticks: 20-ms square laser pulses at 20 Hz for 1 s). Examples of PSTHs from 6 putative AgRP neurons (**A**) and 6 arcuate neurons that were strongly (>20%) and significantly suppressed by AgRP neuron photostimulation (ARC_inh_; **B**). (**C**) Phasic entrainment to individual laser light pulses in AgRP neurons. Examples of cycle histograms (which include firing rates during all pulses from all trials, see ‘Materials and methods’), from four putative AgRP neurons that were phase-locked to light stimulation (light blue). Asterisks denote phases relative to laser pulse onset (time 0) with firing rates that were significantly higher than phase-shuffled control levels (p < 0.0001; see ‘Materials and methods’). **DOI:** http://dx.doi.org/10.7554/eLife.07122.004

We separated ARC neurons into classes by matching spikes with near-identical waveforms obtained during ongoing activity and periods of photostimulation and identifying neurons that were either driven (putative AgRP neurons, henceforth termed ‘AgRP neurons’) or suppressed (‘ARC_inh_ neurons’) by laser photostimulation (Lima et al., 2009, Cohen et al., 2012; see below, Figure 1A–E and ‘Materials and methods’). Across 75 daily sessions in 15 mice, we recorded spiking activity in 100 ARC neurons, of which 41 were optogenetically identified as AgRP neurons on the basis of sustained firing increases during photostimulation (see Figure 1D, Figure 1—figure supplement 1A; see ‘Materials and methods’ for additional details of classification). We also recorded activity of 26 nearby ARC neurons that were significantly and strongly suppressed (by at least 20%) by photostimulation (Figure 1E and Figure 1—figure supplement 1B; see also ‘Materials and methods’). Because the only ARC neurons currently known to be inhibited by AgRP neurons are POMC neurons (Cowley et al., 2001, Atasoy et al., 2012), a large fraction of ARC_inh_ neurons are likely to be POMC neurons. We also recorded from an additional 33 nearby neurons that were unaffected by photostimulation (‘ARC_other_’). All recordings were performed in mice habituated to head restraint (see ‘Materials and methods’), as this enabled recordings with greater stability from a larger number of electrodes.

### Opposing modulations in ARC neuron firing across times of day

**Figure 2.**
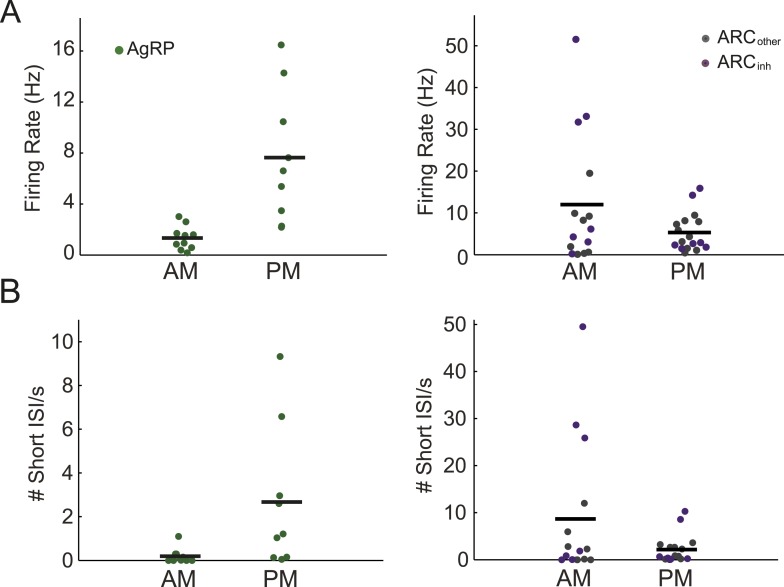
Arcuate neurons demonstrate changes in firing rate across the light period. (**A**) AgRP neurons (green dots) fired significantly more in the afternoon (when caloric deficiency is increased and the dark period is approaching) than in the morning (AM: 1.4 ± 0.3 Hz, n = 10; PM: 7.6 ± 1.7 Hz, n = 9; t-test, p = 0.001), while all other ARC neurons showed the opposite trend (AM: 12.0 ± 4.0 Hz, n = 15; PM: 5.3 ± 1.1 Hz, n = 17; t-test, p = 0.08). ARC_inh_ neurons (purple dots) showed a similar trend (AM: 18.5 ± 7.6 Hz, n = 7; PM: 5.9 ± 2.4 Hz, n = 7; t-test, p = 0.14). Note the presence of ARC_inh_ neurons with very high mean spiking rates above 30 Hz. (**B**) Same plots as in **A**, but displaying the rate of short inter-spike interval events (ISI; spikes occurring <50 ms apart) in morning vs afternoon recordings. AgRP neurons showed a 14-fold increase in short ISI events (AM: 0.03 ± 0.11 Hz; PM: 2.7 ± 1.1 Hz; t-test p = 0.02), while non-AgRP ARC neurons showed a trend toward a decrease in short ISI events in the afternoon (AM: 8.7 ± 3.8 Hz; PM: 2.2 ± 0.7 Hz; t-test, p = 0.08); neurons that were inhibited by photostimulation showed a similar trend (AM: 15.3 ± 7.4 Hz; PM: 2.9 ± 1.7 Hz; t-test, p = 0.13). **DOI:** http://dx.doi.org/10.7554/eLife.07122.005

In a first experiment in ad libitum-fed mice, we measured the firing of ARC neurons during daily 1-hr recording sessions at different phases of the light period, as the stomach is emptying (Kentish et al., 2013), levels of ghrelin, a hormone known to increase AgRP neuron activity, are rising (Tschop et al., 2000, Cummings et al., 2001, Wang et al., 2002, Bodosi et al., 2004), and minimal feeding is occurring as compared to the subsequent dark period (Lu et al., 2002). Stable firing across tens of minutes (Figure 1F) allowed reliable estimation of mean firing rate. As predicted by diurnal variations in in vitro AgRP neuron activity (Yang et al., 2011, Krashes et al., 2013) and in ARC expression of *Agrp* mRNA (Lu et al., 2002), AgRP neurons demonstrated a significant, approximately fivefold increase in firing in afternoon vs morning recordings (p = 0.001; n = 10 vs 9 neurons, respectively; Figure 2A). In contrast to AgRP neurons, we observed a trend towards decreased firing in afternoon vs morning recordings across all non-AgRP neurons (p = 0.09, n = 32 neurons), with ARC_inh_ neurons showing a similar trend (p = 0.13, n = 14; Figure 2A).

**Figure 2—figure supplement 1.**
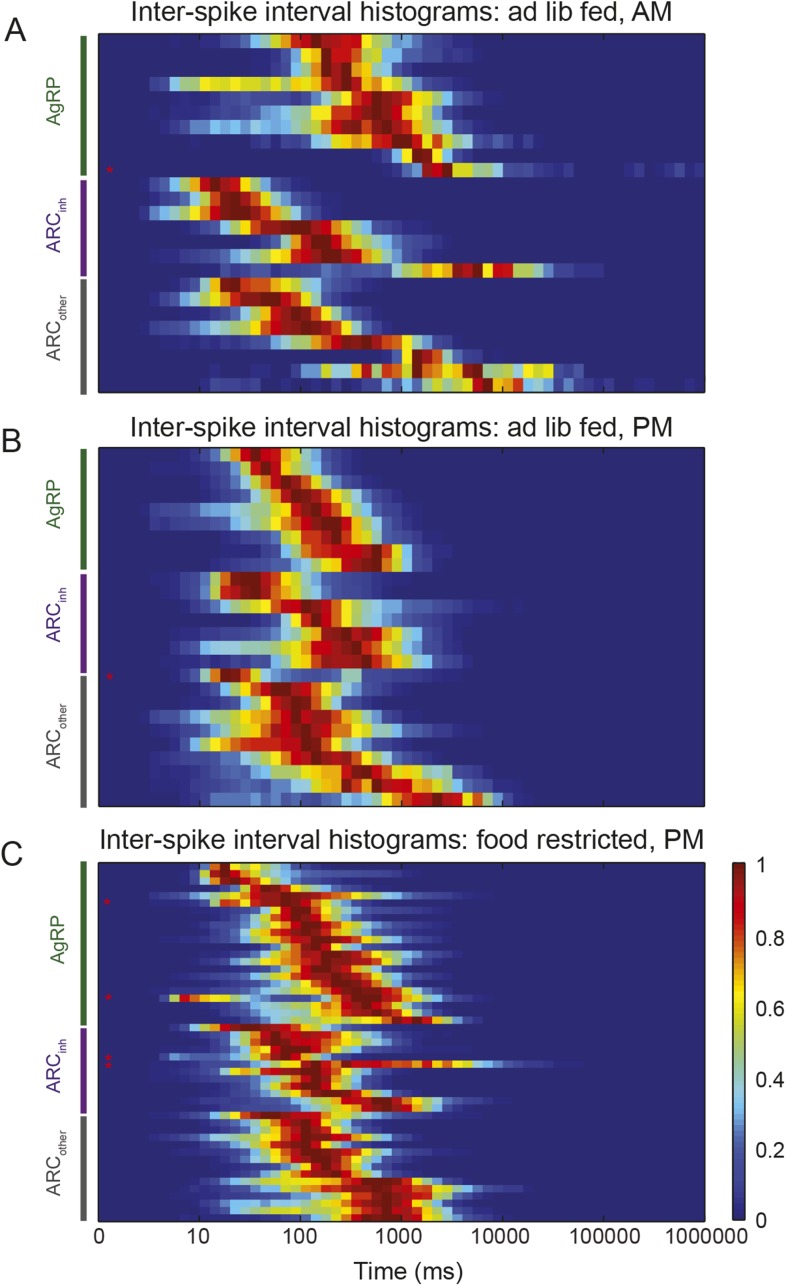
Characterization of ISI statistics in arcuate neurons. Plot of all ISI distributions (each row depicts one neuron) in the ad libitum feeding experiment (**A** and **B**) and in the instrumental conditioning task (**C**). Distributions were grouped by class (green: AgRP; purple: ARC_inh_; gray: ARC_other_). All distributions were normalized by their peak values. Distributions were relatively broad compared to in vitro recordings, as characterized by coefficients of variation (defined as the (mean)/(standard deviation) of the log_10_(ISI) distribution) greater than 1 (coefficient of variation across all neurons recorded in both experiments: AgRP: 1.21 ± 0.07; ARC_inh_: 1.64 ± 0.21; ARC_iother_: 1.51 ± 0.13; all values are mean ± SEM; no significant differences across classes, KS-test, p > 0.05). Firing rate distributions were tested for bimodality using Hartigan's Dip test; in total, 6/100 ARC neurons (red asterisks) had significantly bimodal distributions of ISIs (p < 0.05). **DOI:** http://dx.doi.org/10.7554/eLife.07122.006

AgRP neuron photostimulation of food-seeking and food intake has been shown to be dependent on stimulation frequency, with greater potency at 20 Hz than at 5 Hz or 10 Hz (Aponte et al., 2011). Further, the release of peptides is also likely to depend on spike frequency (Summerlee and Lincoln, 1981, Aponte et al., 2011, Arrigoni and Saper, 2014, Schone et al., 2014). Thus, we sought to gain insight into instantaneous spiking frequency in our sample of ARC neurons by considering the distribution of inter-spike intervals (ISIs). Most neurons (94/100) demonstrated tonic firing with unimodal ISI distributions (Figure 2—figure supplement 1; Hartigan's Dip Test, p < 0.05), suggesting the absence of pronounced burst-like behavior (van den Top et al., 2004). In contrast to in vitro recordings, where spiking often demonstrates machine-like regularity, we observed that ISI distributions in vivo were quite broad (ratio of standard deviation to mean, or coefficient of variation, exceeding a value of 1; see Figure 2—figure supplement 1 for details) for all three classes of neurons, likely reflecting strong moment-to-moment fluctuations in synaptic input in vivo, as well as differences in the cellular milieu in vivo vs. ex vivo. It is notable that, in the context of recordings from AgRP neurons in free-feeding mice, we rarely observed short ISIs (<50 ms) in morning recordings, but observed a roughly 14-fold increase in occurrence of such events in afternoon recordings (Figure 2B; p = 0.02). In contrast, the non-AgRP neurons (ARC_inh_ and ARC_other_) show the opposite trend, with short ISIs occurring more often in the morning than in the afternoon (p = 0.13). Taken together, these in vivo increases in AgRP firing and decreases in ARC_inh_ firing from morning to afternoon recordings are consistent with the homeostatic roles proposed for AgRP and POMC neurons in feeding behavior (Krashes et al., 2011).

### Opposing modulations in ARC neuron firing across tens of minutes during feeding behavior

**Figure 3.**
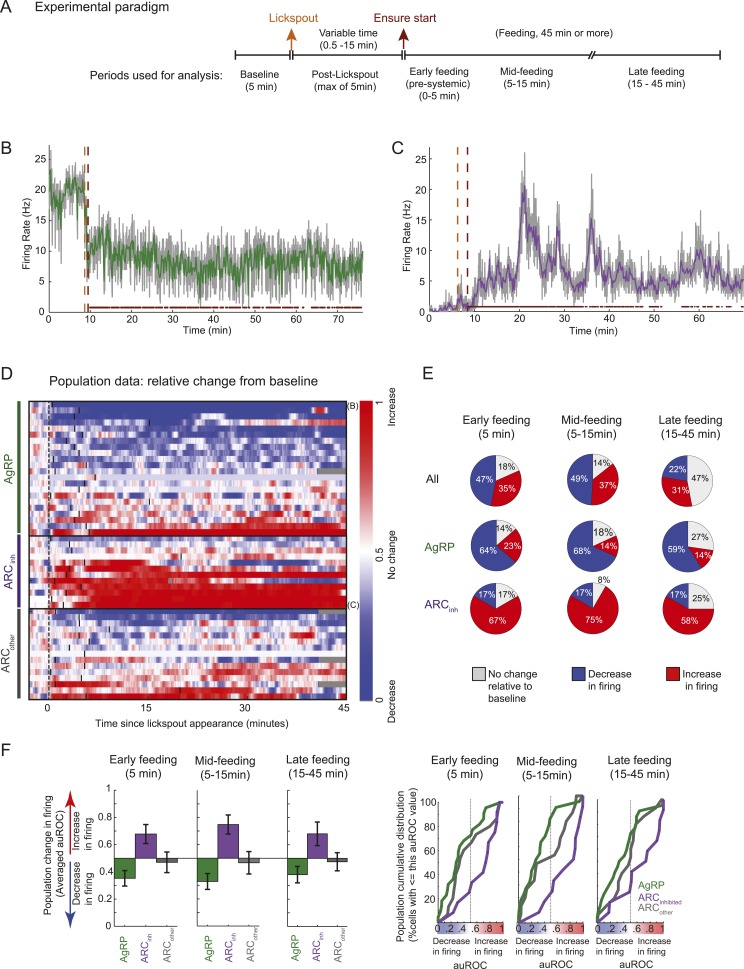
Arcuate neurons are modulated on the timescale of minutes by feeding. Following instrumental conditioning for liquid food rewards (Ensure) in food-restricted mice, we recorded arcuate neuron changes during feeding. (**A**) Experimental paradigm. First, baseline spiking was recorded for at least 5 min. A lickspout was then positioned close to the snout. After a variable duration (0.5–15 min), food was made available, at which point licking resulted in a delivery of 10 μl of liquid food. Typically, the mouse continued to eat for at least 45 min, beginning with almost continuous licking and gradually transitioning to sparser feeding bouts (see below). (**B**) An example AgRP neuron demonstrating a fast and sustained decrease in firing within minutes of presentation of a lickspout (orange vertical dashed line; see also Figure 4A) and access to food (maroon vertical dashed line). Dots above x-axis signify 10-s bins in which licking occurred. Gray trace: firing rate in 2-s bins; colored trace: 10-s bins. Significant decreases in firing were observed from pre-lickspout baseline to the periods following access to food (p < 0.001 for early-, mid-, and late-feeding periods). (**C**) Similar to **B**, but for an example ARC_inh_ neuron that demonstrated significant increases in firing post-feeding onset (p < 0.001, for early-, mid-, and late-feeding periods; see also Figure 4A). (**D**) Timecourses of increases (red), decreases (blue), or no reliable change (white) in firing from pre-lickspout baseline (gray vertical dashed line) for each cell recorded during this task (n = 49). For visualization purposes, this plot employs a normalized index called the area under the Receiver Operating Characteristic Curve (auROC; see ‘Materials and methods’). Short vertical black lines denote the onset of food availability. Example neurons in **B** and **C** are denoted by ‘B’ and ‘C’, respectively. (**E**) Proportion of cells recorded that responded with a significant (two-sample KS-test, p < 0.025) increase (red), decrease (blue), or with no change in firing at 0–5 min (left), 5–15 min (middle), and 15–45 min (right) post-feeding onset. Data include 22 AgRP neurons, 12 ARC_inh_ neurons, and 15 ARC_other_ neurons from 5 mice. (**F**) Comparison of auROC values, across AgRP, ARC_inh_, and ARC_other_ (green, purple, and gray, respectively) neurons, during early-, mid-, and late-feeding periods (left, middle, and right panels, respectively). Left: bar plot showing averaged auROC (a value of 0.5 reflects no change in distributions of firing rate). For early-, mid-, and late-feeding periods, mean auROC for ARC_inh_ (early: 0.68, mid: 0.75, late: 0.68) is significantly larger than those of AgRP (early: 0.35, mid: 0.33, late: 0.38; Analysis of variance, p = 0.046, 0.00004, 0.014, respectively). Error bars denote SEM. Right: cumulative distribution of auROC values across neurons in each class, for all feeding periods. The abscissa value at an ordinate of 50% indicates the median auROC for each class. **DOI:** http://dx.doi.org/10.7554/eLife.07122.007

**Figure 4.**
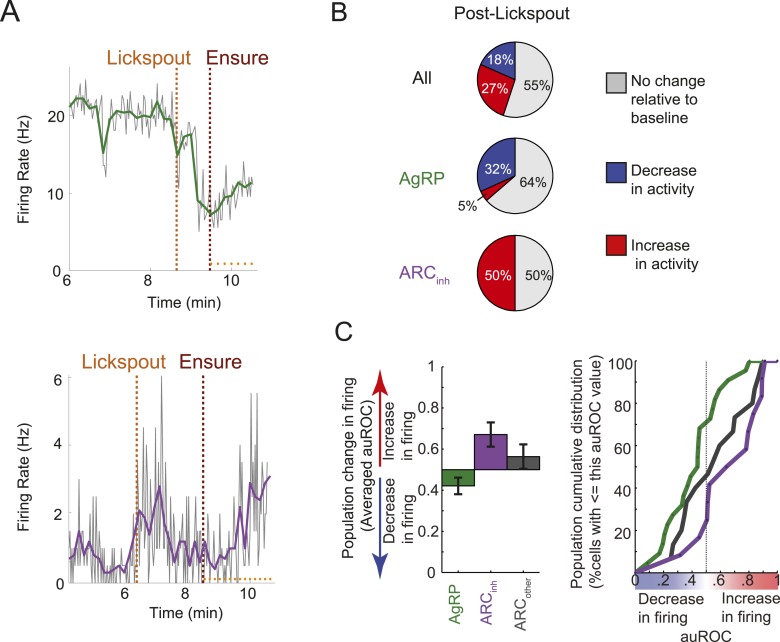
Many ARC neurons are modulated within minutes following food cue presentation, but prior to feeding. (**A**) Spiking activity of the same cells shown in Figure 3B,C, zoomed-in to illustrate the drop in spiking in response to presentation of a food cue (lickspout placement near the snout; orange dashed line) but prior to onset of food delivery (maroon dashed line). Top: AgRP neuron showing a significant food cue-induced decrease (two-sample KS-test, p < 0.001); Bottom: ARC_inh_ neuron showing a significant food cue-induced increase (p < 0.001). (**B**) Proportion of cells recorded that responded with a significant (two-sample KS-test, p < 0.025) increase in firing (red), decrease in firing (blue), or with no change in firing (gray) following lickspout placement but prior to feeding. (**C**) Comparison between averaged auROCs of AgRP, ARC_inh_, and ARC_other_ neurons (green, purple, and gray, respectively) for the period between lickspout placement and feeding (cf. Figure 3F). Left: bar plot of mean auROC (0.5 indicates no change in a neuron's distribution of spike rates) across classes. Mean auROC for ARC_inh_ (0.67) is significantly larger than that of AgRP (0.42) (Analysis of variance, p = 0.0046). Error bars denote SEM. Right: cumulative distribution of auROC values for all ARC classes. **DOI:** http://dx.doi.org/10.7554/eLife.07122.010

In a second experiment, we recorded activity of ARC neurons in food-restricted mice trained to lick a lickspout to receive high-calorie liquid food (Ensure; see ‘Materials and methods’). As described in Figure 3A, during each daily session, we recorded spiking activity (i) prior to presence of any food-predicting cues (‘baseline period’) and (ii) prior to feeding but following the presentation and positioning of the lickspout in front of the snout, and (iii) during a period of at least 45 min in which the mouse could lick to receive food rewards. Across sessions, the onset of food availability had a variable delay following lickspout placement in order to disambiguate ARC responses to initiation of feeding (Figure 3) from any pre-feeding responses to food-associated cues (Figure 4). The main findings are illustrated in two example ARC neurons (Figure 3B,C). We observed a dramatic decrease in firing rate in the AgRP neuron (Figure 3B) when comparing the 5-min baseline period before lickspout placement (orange dashed line) to the 45-min period following onset of food consumption (red dashed line). By contrast, we observed a large increase in firing in the example ARC_inh_ neuron (Figure 3C).

**Figure 3—figure supplement 1.**
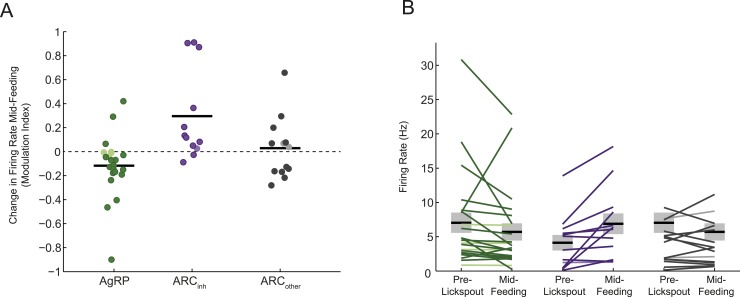
Different feeding effects in separate populations of arcuate neurons. (**A** and **B**) Comparisons of auROC firing modulation index (**A**; modulation index: (5–15 min post ensure—baseline)/(5–15 min post ensure + baseline)) and firing rate (**B**) mid feeding (5–15 min post-feeding onset) as compared to baseline, for AgRP neurons (green), ARC_inh_ neurons (purple), and ARC_other_ neurons (gray). More darkly colored lines represent significantly modulated neurons (KS-test; see Figure 2D). Population changes in absolute firing rate (mean: black horizontal bars; SEM: gray) appeared generally consistent with changes at the level of single neurons (Figure 3B,C) but were significant only for the ARC_inh_ class (AgRP neurons: p = 0.16; ARC_inh_ neurons, p = 0.01; ARC_other_ neurons; p = 0.8; paired sample t-test), likely due to the large variability in baseline firing rates (see main text). **DOI:** http://dx.doi.org/10.7554/eLife.07122.008

**Figure 3—figure supplement 2.**
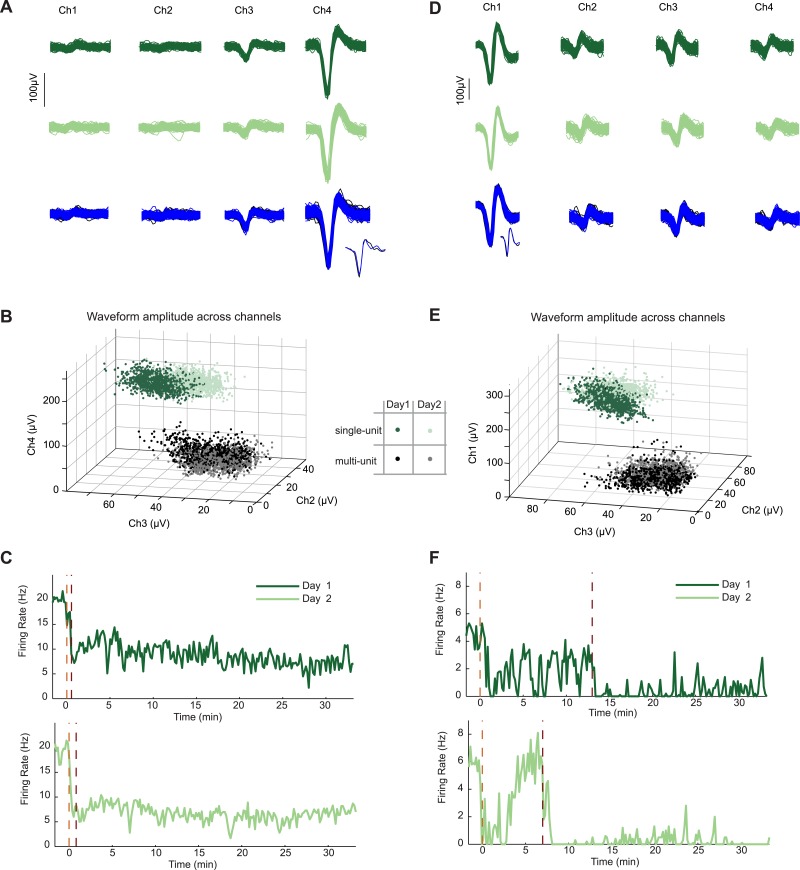
Neurons recorded across multiple days show similar firing changes to food-associated cues and feeding. In two mice, we recorded the same AgRP neuron in two subsequent sessions during the feeding task. (**A**) Similarity of spike waveforms in the four recording channels across day 1 (top) and day 2 (middle). Similarity of spike waveforms (bottom) from prior to (black) and during (blue) AgRP photostimulation. Inset, one individual spike waveform from each epoch. (**B**) Ratio of waveform amplitudes across electrode channels was also similar. (**C**) Spiking activity during day 1 and 2 of recording from the same AgRP neuron demonstrated strikingly similar responses across days to both lickspout placement (orange dashed line) and to the onset of feeding (maroon dashed line). Clear feeding-related drops in firing occurred that were distinguishable from the earlier drops following lickspout placement. (**D**–**F**) Example data from a second AgRP neuron. Same notations as in **A**–**C**. Note partial recovery following initial post-lickspout drop in firing, but not following post-feeding drop in firing. **DOI:** http://dx.doi.org/10.7554/eLife.07122.009

Abrupt and sustained decreases in AgRP neuron firing, and increases in ARC_inh_ neuron firing, were also observed at the population level. We visualized firing changes at the population level as follows: because neurons in our sample exhibited a range of baseline firing rates and firing rate variability (Figure 3—figure supplement 1), we computed an index of reliable changes in firing rate between the baseline period (5 min) prior to lickspout placement, and a 1-min sliding window of time following this baseline period. This index, called the area under the Receiver Operating Characteristic (auROC; see ‘Materials and methods’), has a value of 0 (blue) in periods where a neuron's firing has decreased robustly (i.e., the distribution of firing rates at the tested period is entirely below the firing rate distribution of the baseline period), a value of 1 (red) in periods where firing has increased robustly, and a value of 0.5 for periods with no change in firing (i.e., where the distribution of firing rates in the test period is indistinguishable from baseline). Using this index, we observed that AgRP neurons typically showed a rapid and sustained decrease in firing (Figure 3D) when comparing pre-lickspout baseline firing (prior to gray vertical bar) with firing rates in a sliding window following onset of feeding (black vertical bars). By contrast, ARC_inh_ neurons, a class that likely includes POMC neurons, typically showed a rapid and sustained increase in firing (Figure 3D).

We quantified these feeding-related changes from baseline firing (pre-lickspout placement) in three windows of time: (i) between 0–5 min post-feeding onset, a time that likely precedes most nutrient absorption and counter-regulatory visceral and hormonal changes (e.g., de Araujo et al., 2008), (ii) between 5–15 min post-feeding onset, a time when mice were still licking for food at a maximal rate but when systemic changes may begin to occur (de Araujo et al., 2008), and (iii) between 15–45 min post-feeding onset, a time when licking for food decreased and became more sporadic (see dots above x-axis in Figures 3B,C). During each analysis window, we tested for significant differences in mean firing between baseline and post-feeding firing rates (by K-S tests; using distributions of firing rates from each 5 s bin in each period), and we quantified population estimates of the auROC index described in Figure 3D (which reflects discriminability of firing rate distributions between pre-lickspout and post-feeding periods).

Surprisingly, during early feeding (0–5 min post-feeding onset), mean firing rates were significantly different than baseline in 82% (40/49) of all ARC neurons (Figure 3E). In particular, 64% (14/22) of AgRP neurons had significantly decreased mean firing during this period, while only 23% (5/22) of AgRP neurons had increased mean firing (Figure 3E). The average auROC index across all AgRP neurons was significantly less than 0.5 (i.e., a discriminable decrease in distribution of firing rates; Figure 3F, top left panel; p = 0.016). Indeed, auROC indices for most AgRP neurons were <0.5 during this period, as illustrated in the cumulative distributions plot (green distribution in Figure 3F, bottom left panel). In contrast to these decreases in firing, ARC_inh_ neurons demonstrated opposite changes during this early pre-feeding period, as 66% (8/12) of ARC_inh_ neurons showed a significant increase in firing, while only 17% (2/12) showed a significant decrease (see Figure 3E; mean auROC significantly *above* 0.5, p = 0.01, see Figure 3F).

These drops in firing rate in AgRP neurons, and increases in ARC_inh_ neurons, generally persisted across later feeding periods (Figure 3E,F; see also Figure 3—figure supplement 1). In general, feeding-induced changes from pre-lickspout baseline period remained consistent but began to abate by 15–45 min post-feeding onset, a time of decreased licking and consumption (Figure 3E; see also Figure 3F, bottom panels; 53% of all 49 ARC neurons showed significant changes in mean firing at 15–45 min after feeding onset, vs 82% and 86% of ARC neurons during earlier post-feeding periods). Notably, AgRP neurons with higher initial firing rates prior to lickspout placement were more likely to show a larger subsequent drop in firing after feeding (e.g., at 5–15 min post-feeding onset: r = −0.54; p = 0.0091; Figure 3—figure supplement 1B). It is possible that certain AgRP neurons may have already decreased their activity prior to the start of recording, due to other contextual food-associated cues appearing at each session's onset. As such, our findings likely provide a conservative estimate of the number of AgRP neurons with cue- and food-related decreases in firing (see ‘Discussion’).

In general, these findings demonstrate clear and opposite changes in spiking activity in the majority of AgRP and ARC_inh_ neurons during feeding behavior, consistent with recent studies measuring changes in calcium activity in ARC neurons during feeding (Betley et al., 2015, Chen et al., 2015). In addition to these changes in firing, our data provided additional information regarding absolute activity levels following the onset of feeding. Interestingly, while feeding reduced firing rates, it did not abolish firing in AgRP neurons, and firing rates remained elevated relative to certain ad libitum conditions. Specifically, we found that the spiking rate of AgRP neurons recorded in food-restricted mice following refeeding (15–45 min after onset of feeding, whether during epochs that involve or do not involve licking, 5.9 ± 1.3 and 5.8 ± 1.3 spikes/s, respectively; N = 22 neurons) was significantly higher than firing rates measured in free-feeding mice at the onset of the light cycle following night feeding (1.4 ± 0.3 spikes/s; N = 10; p = 0.03 in both cases). These data suggest that the early, feeding-related drop in AgRP neuron activity represents a partial drop in firing that does not reach the lower levels of firing exhibited by these neurons in the absence of caloric deficiency.

### Opposing modulations in ARC neuron firing by food-associated cues *prior* to ingestion of food

We next assessed the possibility of even earlier changes in firing, immediately after presentation of the food-predicting cue (lickspout placement near the snout) but prior to onset of feeding. As shown in Figure 4A,B (zoomed-in plots of same example AgRP and ARC_inh_ neurons as in Figure 3B,C), a closer inspection of this period (between the orange and maroon dashed lines) suggests that ARC firing rates may begin to change from baseline *before* feeding has even started, likely due to the presentation of the food-predicting cue (lickspout placement). Indeed, almost half of all ARC neurons recorded (45%, 22/49; 2 sample KS-test, p < 0.025) showed significant changes in mean firing in the 5-min period following lickspout placement, but prior to feeding. As with post-feeding changes, 32% (7/22) of AgRP neurons showed a significant ‘anticipatory’ drop in firing, while only 5% (1/22) showed a significant increase. By contrast, 50% (6/12) of ARC_inh_ neurons showed significant anticipatory *increases* in firing, while none showed a significant drop. Similar effects were evident in the auROC change index, plotted in Figure 3D (in the period following the black vertical dashed line, but prior to the black vertical bar in each row) and quantified in Figure 4C. Thus, a large subset of AgRP neurons decrease spiking activity in the minutes following presentation of a food-predicting cue, while ARC_inh_ neurons showed the opposite effect. These findings suggest that AgRP neurons decrease their firing upon identification of an upcoming source of food, either to decrease the drive to continue the search for food (see ‘Discussion’), or in anticipation of future meal-induced restoration of caloric deficit.

### Reliability of AgRP neuron modulation by food cues and feeding across sessions

While we ensured that neurons recorded across daily sessions were distinct (by electrode adjustment and analysis of spike waveforms), in two cases, we did record from what appeared to be the same AgRP neuron in back-to-back sessions (including the example neuron in Figures 3B, 4A; based on similarity of waveforms and amplitudes across channels of the same tetrode across days, see Figure 3—figure supplement 2A,B,D,E). These examples illustrate that our findings of rapid decreases in AgRP neuron firing, both prior to and following the onset of feeding, were highly consistent for the same neuron when assessed across days. Interestingly, the second example neuron (Figure 3—figure supplement 2C,F) was recorded during two sessions involving long imposed durations between lickspout placement and commencement of feeding (orange and maroon dashed vertical lines). On both days, this AgRP neuron showed a transient drop following lickspout placement that partially recovered over minutes, followed by a more sustained drop following onset of feeding (Betley et al., 2015, Chen et al., 2015). Thus, such stable recordings of the same ARC neurons across days should be possible using our approach (see e.g., Kentros et al., 2004, Siegle and Wilson, 2014, Thorn and Graybiel, 2014) and can provide valuable insights into slow and fast motivational changes.

### AgRP photostimulation results in increased licking and food consumption

**Figure 5.**
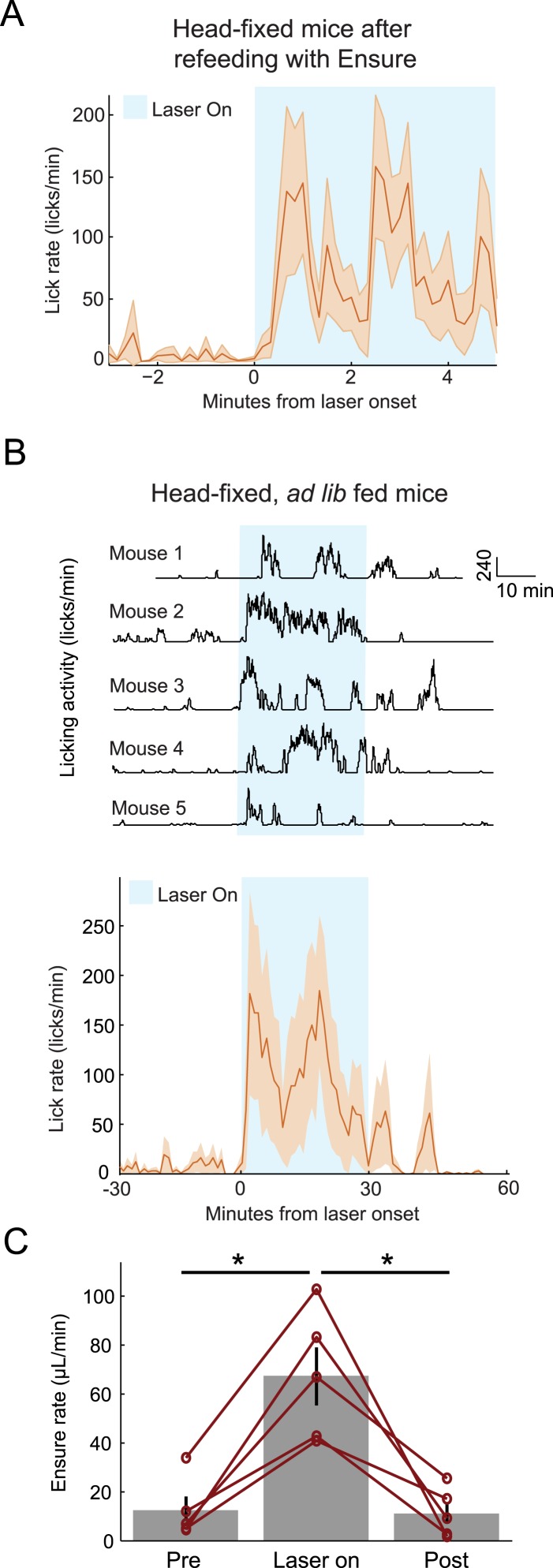
Optogenetic activation of AgRP neurons promotes licking behavior and food consumption in head-restrained mice. (**A**) Mice that have been fed Ensure to satiety (see Figures 3, 4) subsequently increased licking for Ensure in response to optogenetic photostimulation of AgRP neurons. (**B**) Ad libitum-fed mice also increased licking for Ensure in response to optogenetic photostimulation of AgRP neurons, even when head-restrained (Top: individual single-session examples from 5 separate mice; Bottom: mean ± SEM of lick rate from 5 mice). (**C**) Ad libitum-fed mice increased food consumption in response to optogenetic stimulation of AgRP neurons, even when head-restrained (asterisks indicate paired t-tests, p<0.02). **DOI:** http://dx.doi.org/10.7554/eLife.07122.011

Optogenetic stimulation of AgRP neurons drives voracious feeding (Aponte et al., 2011). Previous studies, however, were performed in freely moving mice with access to solid food. We confirmed that AgRP photostimulation in a headfixed mouse trained to lick for liquid Ensure also drives feeding. First, at the end of feeding experiments (described above), when mice were sated (defined by voluntary abstinence from licking for liquid food), we repeated the photostimulation procedure to confirm identification of recorded cells. Here, we investigated whether this AgRP photostimulation would induce additional licking for liquid food (n = 11 sated sessions from 4 mice, including all sessions in which the mouse did not lick for Ensure in the 3 min preceding laser stimulation onset). Mice showed a marked increase in licking behavior after photostimulation onset compared to the period preceding photostimulation (Figure 5A; p = 0.03). In a separate experiment, we habituated another cohort of mice to head-fixation and trained them (for 1–2 days, under mild food restriction) to lick for Ensure, followed by at least 5 days with ad libitum access to food. Under these free-feeding conditions, which more faithfully approximate published data on AgRP-driven feeding behavior (Aponte et al., 2011, Krashes et al., 2011), mice consistently increased licking behavior and food consumption in response to AgRP photostimulation (Figure 5B,C; pre-photostim.: 12 ± 2 μl/min; during photostim.: 67 ± 5 μl/min; post-photostim.: 11 ± 2 μl/min, mean ± SEM across 5 mice; pre-photostim vs during photostim: p = 0.004, during photostim vs post-photostim: p = 0.015; paired t-test).

### Fast modulations in ARC neurons firing within 1 s of a lick or lick bout

**Figure 6.**
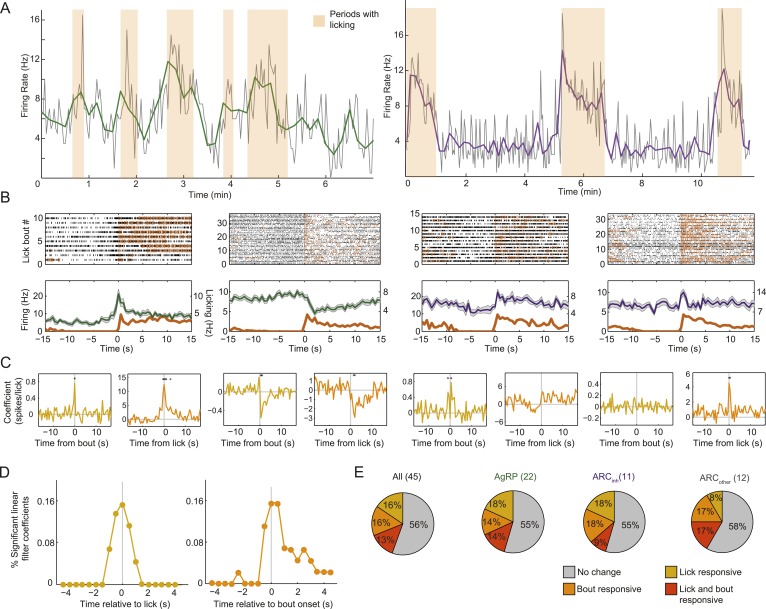
Arcuate neurons are modulated on short timescales by licking activity. We evaluated whether ARC neuron firing could be modulated by bouts of licking for food (defined by >8 s without licking, followed by a burst of >3 licks) and/or by the occurrence of an individual lick (timescale of <1 s). (**A**) Example firing rate traces (gray: 2-s bins; green/purple: 10-s bins) and licking (orange) from an AgRP neuron (left) and an ARC_inh_ neuron (right) that both showed positive correlations between firing rate and licking bouts. (**B**) Top: raster plots of spiking (black ticks) and licking (orange ticks) for four example neurons, aligned to the onset of a licking bout. Bottom: average firing (mean ± SEM) relative to bout onset for AgRP neurons (green) and ARC_inh_ neurons (purple). Dark orange traces are average lick rates. Note that firing of some cells appears linearly related to frequency of individual licks (e.g., third panel from left), while firing in other cells appeared more strongly modulated by bout onset (first and fourth panles from left). Several AgRP neurons (e.g., second panel from left) showed a reliable decrease in firing at bout onset. (**C**) We estimated the degree of modulation of firing across time relative to an individual lick (gold traces), and relative to an individual bout onset (orange), using multiple linear regression. Asterisks indicate times of significant modulation, relative a lick or lick bout (F-test, p < 0.002, corrected for multiple comparisons; see ‘Materials and methods’). (**D**) Population distribution of times relative to a single lick (gold) or lick bout (orange) at which significant modulation of firing (asterisks in **C**) occurred across 44 cells. Note that many neurons began changing their firing *before* the onset of a lick or lick bout (gray vertical lines), and that modulation mostly occurred within ±1–2 s of onset of a lick or lick bout, demonstrating modulation of ARC neuron firing at a surprisingly fast timescale. (**E**) Proportions of neurons in each class that were significantly modulated by individual licks (gold), lick bouts (orange), both (red), or neither (gray). **DOI:** http://dx.doi.org/10.7554/eLife.07122.012

These feeding-related changes at the timescale of minutes led us to ask whether ARC neurons can be modulated at the even faster timescale of seconds, by individual licks and/or by bouts of licking preceded by several seconds without licking (see Figure 6; Chen et al., 2015). If such modulation existed, it could imply that AgRP neurons and other ARC neurons might contribute to momentary fluctuations in the drive to seek and/or consume food, even at the level of licking microstructure (Davis, 1989).

**Figure 6—figure supplement 1.**
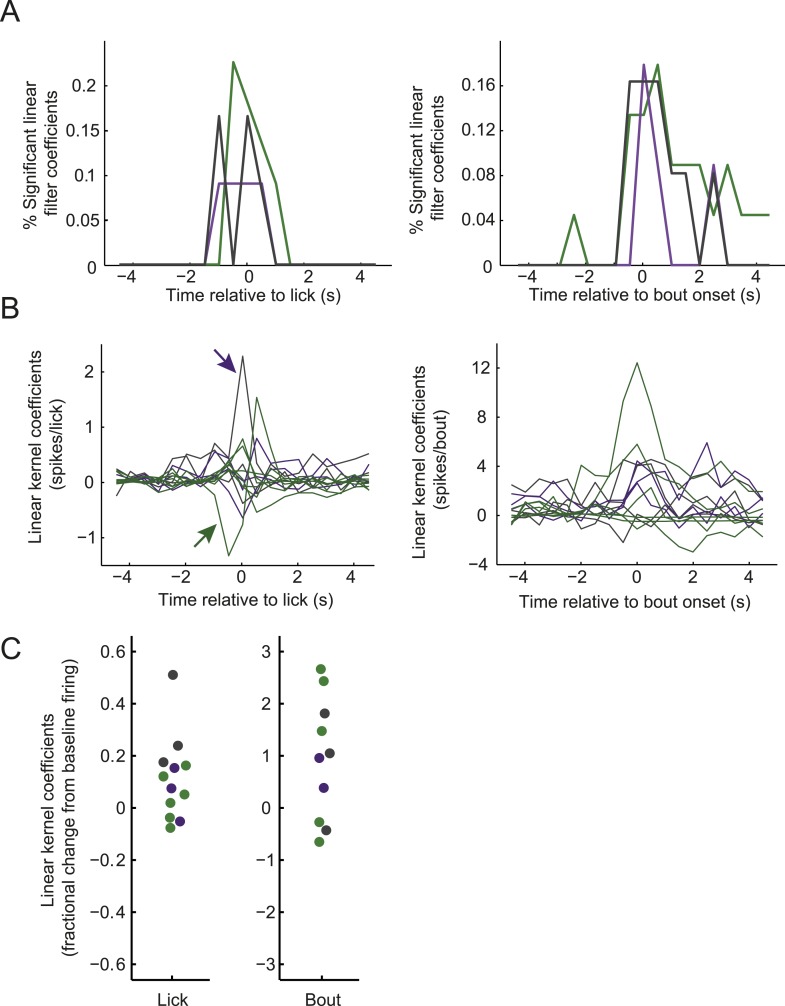
Modulation of firing by licking across classes of arcuate neurons. (**A**) Distribution of times relative to lick onset (left) or lick-bout onset (right) where ARC neuron firing was significantly modulated, as in Figure 3D but plotted separately for AgRP neurons (green), ARC_inh_ neurons (purple), and ARC_other_ neurons (gray). (**B**) Linear relationship (kernel estimate from multiple linear regression, see ‘Materials and methods’) of ARC firing rate modulation (in 0.5-s bins) surrounding lick onset (left) or lick-bout onset (right), as in Figure 3C but plotted for all neurons exhibiting significant modulation for at least one time point (F-statistic for regression, p < 0.002, corrected for multiple comparisons across time points). Colors indicate cell class, as in **A**. The left panel indicates, for example, that a putative POMC neuron (purple arrow) increased its firing, on average, by approximately 2.4 spikes in the 0.5-s bin at which an individual lick occurs, while an AgRP neuron (green arrow) decreased its firing, on average, by 1.3 spikes in the 0.5 s *prior* to the occurrence of each individual lick. (**C**) To provide an estimate of the sign and relative magnitude of the modulation of ARC firing by lick events, we estimated the time point (within ±2 s of the lick or bout onset) with the larger significant modulation coefficient, for all traces in **B**. We then normalized this coefficient to the mean firing rate of the cell (offset estimate from regression analysis) to provide an estimate of relative modulation of firing by licking for individual cells. The data show that licking and onsets of lick bouts could decrease or, more often, increase ARC neuron firing, with lick bouts increasing firing by up to ∼300% (three fold), and individual licks by up to ∼50%. **DOI:** http://dx.doi.org/10.7554/eLife.07122.013

**Figure 6—figure supplement 2.**
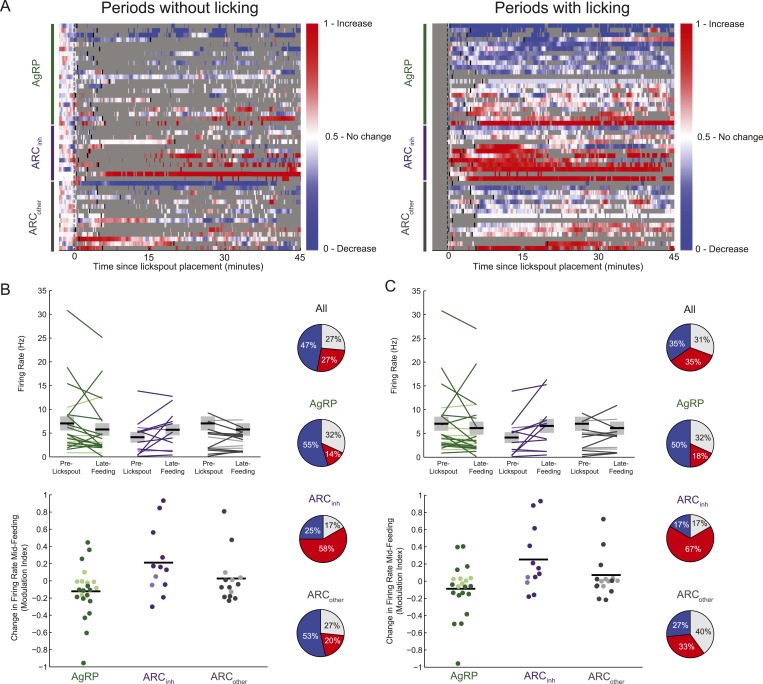
Feeding-related effects are observed independent of licking behavior. (**A**) As in Figure 3D, timecourses of increases (red), decreases (blue), or no reliable change (white) in firing from pre-lickspout baseline (gray vertical dashed line) are plotted for each cell recorded during this task. For visualization purposes, this plot employs a normalized firing index called the auROC; see ‘Materials and methods’. Short black lines denote the onset of food availability. In the left panel, all time bins within 5–10 s of any lick are grayed out, to better visualize feeding-related responses not contaminated by licking effects. In the right panel, all firing changes data surrounding any lick events are plotted. Note that sustained decreases (blue) in AgRP neuron firing, and increases (red) in ARC_inh_ firing, appear consistent irrespective of licking behavior. (**B**) Comparisons of absolute (top) and normalized (bottom) firing rates, in the 5-min pre-lickspout placement, compared to 15–45 min post-feeding onset, calculated using only periods without licking activity. More darkly colored lines and circles represent significantly modulated neurons (KS-test). Population changes in firing (mean: black horizontal bars; SEM: gray) were not significant for any class (AgRP neurons: p = 0.15; ARC_inh_ neurons, p = 0.11; ARC_other_ neurons; p = 0.37), likely due to within-class variability in baseline firing. Pie charts (right) demonstrate the number of neurons with increased (red), decreased (blue), or no significant change (gray) in activity pre-lickspout compared to 15–45 min post-feeding onset including only periods without licking. (**C**) Same as **B**, but only including periods containing licking activity. In this case, mean firing was significantly increased in ARC_inh_ neurons (p = 0.04), while mean changes in other cell classes were not significant (AgRP: p = 0.31; ARC_other_: p = 0.64). **DOI:** http://dx.doi.org/10.7554/eLife.07122.014

Modulation of firing by bouts of licking (defined as periods of intense licking for food, preceded by at least 8 s without licking) is clearly evident in the example neuron in Figure 6A. We focused analyses on the period from 15 to 45 min post-feeding onset, when initiation of licks and lick bouts was more sporadic (see Figure 3). Firing activity aligned to each lick bout for this and three other neurons (see spike raster plots and average peri-bout time histograms in Figure 6B) revealed that some neurons appear particularly modulated at lick bout onset, while others appear to change their firing proportionally to the number of individual licks at any time (third example from left). We estimated whether each neuron was significantly modulated by licks and/or by lick bouts, using multiple linear regression between timecourses of firing rate, licking, and lick bouts (using 0.5-s bins; see Figure 6C). For the four example neurons, yellow traces in Figure 6C (left panels) show changes in firing rate relative to the moment that a lick occurred (asterisks denote significant firing modulation, F-test, p < 0.002, corrected for multiple comparisons). Similarly, orange traces in Figure 6C (right panels) show changes in firing relative to onset of a bout of licking for liquid food.

At the population level, almost half of ARC neurons were significantly modulated by individual licks and/or lick bouts (Figure 6D,E), with most significant lick- or bout-induced changes in firing (asterisks in Figure 6C) occurring approximately 1 s before and/or after lick/bout onset (Figure 6D and Figure 6—figure supplement 1A,B). The modulation of firing at times substantially preceding the onset of a lick or lick bout suggests that these changes do not only reflect efference motor copies but could partially help drive initiation of upcoming licks. These analyses show that firing rates could be influenced by licking events. However, this rapid source of modulation did not influence the main conclusions in Figure 3 regarding feeding-related decreases in firing in most AgRP neurons, or increases in firing in most ARC_inh_ neurons. These findings remained intact when considering only epochs with or without lick events (Figure 6—figure supplement 2). Feeding-related changes in firing near the end of the meal persisted in the absence of any licking or consumption, suggesting that these changes do reflect actual persistent changes in motivational drive.

Of the 5 AgRP neurons that were significantly modulated by lick bouts, 2/5 showed reliable decreases in firing within seconds of the onset of a licking bout (Figure 6B,C, second neuron from left), similar to the decrease in firing observed over the course of several minutes during the initial onset of feeding behavior (Figure 3). While the rest of our small sample of significant bout-modulated AgRP and ARC_inh_ neurons tended to increase their firing at the time of bout onset (AgRP: 3/5 increased, ARC_inh_: 2/2 increased; Figure 6—figure supplement 1C), future studies will be required to confirm whether populations of AgRP vs ARC_inh_ neurons, on average, also show opposite, lick-related changes in firing at the 1-s timescale.

### Modest correlations in firing at the 1-s timescale, within and across classes of ARC neurons

**Figure 7.**
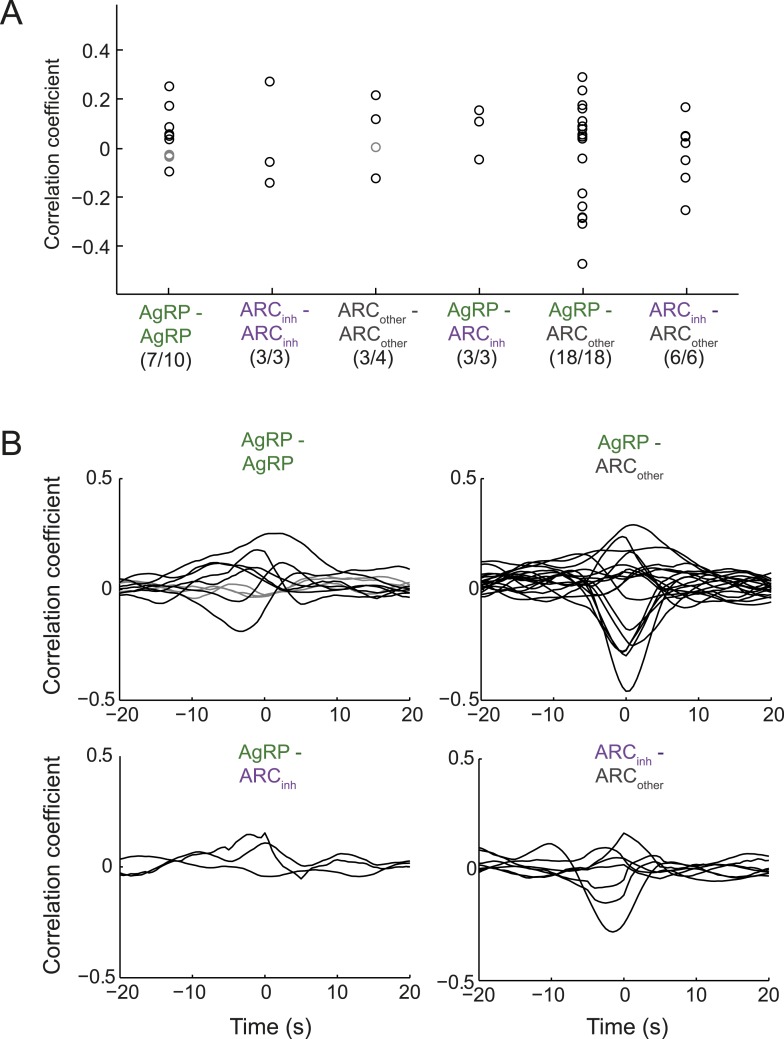
Endogenous correlations between simultaneously recorded pairs of arcuate neurons. (**A**) Correlation coefficients (at zero time-lag) across pairs of simultaneously recorded neurons within a class or across classes of ARC neurons. While 7/10 pairs of AgRP neurons showed significant correlation coefficients (p < 0.05), the correlations were modest (all <0.3). Similar results were observed for other pairs. To ensure that correlation coefficients did not simply reflect slow concurrent changes in firing across neurons, we first removed slow trends in firing from each cell's spike-rate timecourse (slower than ∼100 s, by high-pass filtering firing rate timecourses above 0.01 Hz). (**B**) To examine the timescale of correlation between pairs of neurons, we calculated correlations between pairs at lags up to ±20 s. Most pairs with significant correlations (black lines) peaked near zero time-lag, with correlations falling off by 5 s of lag. These data suggest that pairs of ARC neurons within and across classes can show modest but significant correlations or anti-correlations at the timescale of ∼1 s. **DOI:** http://dx.doi.org/10.7554/eLife.07122.015

One potential means to gain additional insight into the question of whether AgRP and ARC_inh_ neurons show opposite changes in firing at the 1-s timescale, and whether AgRP neurons show synchronized activity at this timescale, is to evaluate simultaneously recorded pairs of neurons. Overall, we found significant but modest pairwise correlations in firing rate, at the 1-s timescale, in 40/44 pairs of simultaneously recorded ARC neurons (see Figure 7A,B and legend). While 3/3 paired recordings from a simultaneously recorded AgRP neuron and ARC_inh_ neuron showed significant correlations, correlation coefficients were modest (Figure 7A, <0.2). Similarly, while 7/10 pairs of AgRP neurons showed significant correlations, these were also quite modest (mostly <0.2). Thus, the correlations in endogenous activity across ARC neurons differ significantly from the hypersynchronous correlations at this timescale that likely occur across ARC neurons during periodic, 1-s duration photostimulation often employed in vivo (Figure 1—figure supplement 1).

## Discussion

**Figure 8.**
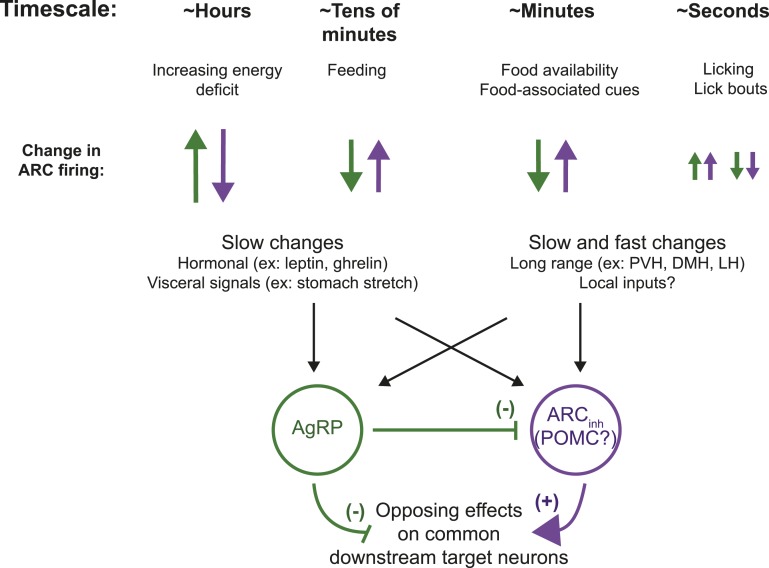
ARC neurons are modulated on multiple timescales. We observed slow, likely homeostatic, changes in ARC neuron activity, consistent with the established role for ARC AgRP and pro-opiomelanocortin (POMC) neurons in regulating energy balance. However, we also demonstrated fast changes, on the order of minutes and even seconds, in response to feeding, food-associated cues, and licking behavior. Differential modulation of these opposing populations of neurons on these timescales may enable both homeostatic and more rapid adjustment of downstream circuits that underlie complex feeding-related behaviors, including food-seeking and food consumption. PVH: paraventricular hypothalamus; DMH: dorsomedial hypothalamus; LH: lateral hypothalamus. **DOI:** http://dx.doi.org/10.7554/eLife.07122.016

In this study, we have developed a means for stable extracellular recordings from individual neurons in the arcuate nucleus of the hypothalamus in awake mice. Using this approach, we demonstrated dynamic changes in spiking activity in classes of ARC neurons across multiple timescales (Figure 8).

First, consistent with in vitro studies, we found an increase in spiking of putative AgRP neurons from morning to afternoon recordings in ad libitum-fed mice (Figure 2), despite the fact that food intake in mice is minimal during this period (Lu et al., 2002). Because the observed increase in endogenous afternoon firing in AgRP neurons does not drive feeding, additional circuits may exist downstream of AgRP neurons (Figure 8) that prevent significant daytime feeding until dark period onset, at which point this ‘drive state’ is released and food-seeking and feeding rapidly occur (Lu et al., 2002). The slow changes in firing that we observed across the light cycle as caloric deficit increases are consistent with prior studies employing indirect measures of AgRP activity (Lu et al., 2002, Ellis et al., 2008), as well as with in vitro recordings from AgRP neurons across times of day (Yang et al., 2011, Krashes et al., 2013). Similarly, the sustained decrease from baseline spiking in AgRP neurons at 15–45 min following onset of feeding (Figure 3) was consistent with previous studies reporting a decrease in AgRP cFos activity 2 hr after scheduled refeeding (Tan et al., 2014) or after post-fast refeeding (Becskei et al., 2009).

Second, in the context of the instrumental feeding task in food-restricted mice, our direct measurement of absolute spiking rates revealed that while spiking in AgRP neurons is reduced following refeeding, even in epochs late in the meal when mice are not licking for liquid food, firing persists and remains elevated (by approximately four fold) as compared to low levels of spiking observed in AgRP neurons recorded from ad libitum-fed mice in the early stages of the light cycle (∼5.9 ± 1.3 spikes/s vs *∼*1.4 ± 0.3 spikes/s). These data further support the notion that additional circuits downstream of AgRP neurons may prevent additional feeding, even in contexts where AgRP neurons continue to fire at intermediate spike rates. Moreover, these data suggest that the rapid decrease in AgRP firing, associated with food cues and meal-initiation (Figures 3, 4; Betley et al., 2015, Chen et al., 2015), may be separable from the firing that persisted at intermediate spiking rates during the meal, which may be associated with homeostatic signals of residual negative energy balance.

We found that AgRP neurons responded with rapid and persistent decreases in activity to both a food-predicting cue (lickspout placement near the snout) and to feeding onset, and that such decreases were sustained at later periods (e.g., 15–45 min post-feeding onset). In contrast to AgRP neurons, ARC_inh_ neurons—including putative POMC neurons—responded with fast and persistent *increases* in firing to both food-predicting cues and food reward. Furthermore, acute modulation of ARC firing rates even occurred down to a timescale of approximately 1 s. Such seconds-long modulations could occur prior to, as well as during, individual licks or bouts of licking for food, which themselves elicited a transient decrease in firing in several AgRP neurons. These fast changes in firing that we observed on the timescale of minutes to seconds in classes of ARC neurons were surprising, as these neurons were presumed, until recently, to be mainly driven by slow homeostatic signals (Lu et al., 2002, Ellis et al., 2008, Kim et al., 2014). However, as reviewed by Berridge 2004, many eating behaviors may ‘co-opt or pre-opt the cue-depletion detectors that trigger hunger in emergency cases of real deficit’.

In the case of AgRP neurons, initial evidence of non-homeostatic influences was suggested based on observations of a decrease in AgRP neuron c-Fos expression 2 hr after consumption of a calorie-free meal (Becskei et al., 2009), though limited temporal resolution and an uncertain relationship between c-Fos and in vivo firing limit the interpretation of these data. More recently, in vivo fiber photometry was used to monitor bulk changes in population calcium activity, pooled from many ARC AgRP neurons (Chen et al., 2015), while another recent study used epifluorescence measurements of calcium activity in individual AgRP neurons in awake mice (Betley et al., 2015). Consistent with our spiking data from individual neurons, these studies observed fast reductions in AgRP neuron calcium activity (within <1 s) during presentation of food-predicting cues and of food (Betley et al., 2015, Chen et al., 2015). Taken together, our direct measurements of spiking activity in AgRP neurons are generally consistent with recent findings observed using measurements of calcium activity in the ARC (see below for further comparison of these different technical approaches).

The full complement of feeding-related behaviors includes sensing caloric deficiency, initiation of food-seeking, consummatory activity, and, ultimately, cessation of feeding. While manipulations of several different brain areas can produce initiation or cessation of food consumption, it remains unknown whether neurons exist whose endogenous in vivo spiking activity parallels the slowly increasing and rapidly terminating drive state associated with food seeking. The motivational drive to find food grows during periods of increasing energy deficit (Saper et al., 2002). During appropriate contexts, this drive should bias actions towards food-seeking, as demonstrated in rodents with increased exploration at the time of scheduled feeding (Moran and Tamashiro, 2007, Mistlberger, 2009, Tan et al., 2014). This behavior persists until a source of food is secured, at which point seeking must stop so that feeding can begin (Craig, 1917, Berridge, 2004). Our findings of slow diurnal increases and fast feeding-related decreases in AgRP neuron firing support the hypothesis that AgRP neurons may also encode a food-seeking drive that can be transiently shut off when food becomes available (Tan et al., 2014), possibly to avoid further ‘appetitive phase’ food-seeking in lieu of consummatory behavior (Craig, 1917, Berridge, 2004). Similar to our recordings in AgRP neurons, previous studies involving a neural circuit that regulates drinking reported rapid *reductions* in firing of vasopressin-secreting supraoptic neurons during drinking of water (Arnauld and du Pont, 1982, Stricker and Hoffmann, 2007). These findings suggest that rapid reductions in firing of neurons promoting specific motivational drives during consumption may be a general mechanism. It is also possible that the fast modulation in spiking activity represents the anticipation of future meal-induced restoration of caloric deficit. To further distinguish between contributions of AgRP neurons to appetitive, consummatory, and/or anticipatory feeding behaviors, it will be instructive, in future studies, to (i) employ larger delays between delivery of food rewards (Cohen et al., 2012; currently inter-reward interval was ≥ 2.5 s), (ii) use lever pressing rather than instrumental licking in order to distinguish requests for food from food consumption (Histed et al., 2012), and (iii) use virtual reality methods in head-fixed mice (Dombeck et al., 2007) to study simulated foraging activity with precise behavioral control and monitoring. Our demonstration that AgRP neuron activity recorded in awake, head-restrained mice shows similar changes as observed in freely moving mice (Betley et al., 2015, Chen et al., 2015), and that activation of AgRP neurons in head-fixed mice has similar effects on consummatory behaviors as previous studies in freely behaving mice (Aponte et al., 2011, Krashes et al., 2011), sets the stage for using head-fixation as a means to probe this feeding circuit during well-controlled behaviors with precise behavioral monitoring, large numbers of trials, and easier experimental access to stable, dense recordings using electrophysiology or two-photon calcium imaging with fewer weight restrictions.

In contrast to AgRP neurons, ARC_inh_ neurons, a group that likely includes POMC neurons that exert opposite effects to AgRP neurons on common downstream targets, demonstrated concomitant *increases* in firing (Figure 8) that may act synergistically with decreases in AgRP firing to more effectively drive these common targets. The recent observations of a rapid increase in population POMC calcium activity in response to food cues, to sustained feeding, as well as during brief individual bouts of licking of liquid food (Chen et al., 2015) are consistent with our spiking data in ARC_inh_ neurons. Optogenetic photostimulation of POMC neurons results in reduced food consumption, although this effect is only evident at longer timescales (Aponte et al., 2011, Zhan et al., 2013). Our data suggest that at least some POMC neurons may act on faster timescales to reduce the drive to seek food. It remains an open question whether these opposing changes in firing are primarily driven by direct inhibition by AgRP neurons (Cowley et al., 2001, Atasoy et al., 2012), or by additional sources of fast input to these neurons (see above). Future studies can begin to elucidate which of the local and long-range sources of fast synaptic input to AgRP and POMC neurons (Figure 8; Krashes et al., 2014) are responsible for various fast and slower changes in firing that we have observed, using optetrode recordings from these inputs as well as by combining ARC recordings with methods for selective silencing of each source of input.

The current study benefited from single-spike resolution to monitor spiking of AgRP neurons in awake mice, down to a timescale of milliseconds. This allowed sub-second measurements of correlations between ARC neuron firing and lick microstructure, as well as sub-second correlations in firing between ARC neurons. Optetrode methods present some challenges, particularly in the ARC: the optetrode (∼300 μm in diameter, with 4–8 70 μm diameter tetrodes extending into the ARC) must penetrate approximately 5.5 mm into the brain to gain access to the relatively small ARC nucleus, likely resulting in lower yield. Similar to previous optetrode studies (e.g., Cohen et al., 2012), some actual AgRP neurons may be classified as ARC_other_ neurons in cases of insufficient photostimulation. While a previous study found that AgRP neurons almost exclusively decrease their calcium activity upon food presentation (Betley et al., 2015), we only observed significant decreases in spiking in approximately two-thirds of AgRP neurons and noted increases in firing in 14–23% of AgRP neurons. These differences may reflect real heterogeneity across AgRP neurons, as previous studies have demonstrated anatomically and functionally distinct populations of AgRP neurons in the ARC (Betley et al., 2013). Alternatively, experimental differences (e.g. different states of baseline meal expectation across paradigms) and/or a possible misclassification of a small number of non-AgRP cells as AgRP neurons could explain the heterogeneity in our data (for additional discussion, see ‘Materials and methods’). As discussed above, recordings of GCaMP6 activity in populations of ARC neurons (Chen et al., 2015) and single cells (Betley et al., 2015) provide powerful complementary approaches to the method described here. Recordings using calcium indicators allow chronic monitoring of calcium activity in AgRP neurons, albeit with some caveats including temporal resolution, estimation of relative contributions of spike-evoked vs intracellular release of calcium, and the potential for neuropil contamination during single-cell recordings. By contrast, our current approach provides robust sensitivity to single action potentials, information regarding inter-spike interval structure of tonic vs bursting ARC neurons, and the capacity to record from both AgRP neurons and other ARC neurons simultaneously. In addition, our recordings of absolute spiking levels (in contrast to relative changes in calcium activity) revealed differences in baseline firing across times of day and across satiety states following refeeding as compared to free-feeding conditions. Together, these mutually informative studies demonstrate consistent changes in the activity of classes of ARC neurons across timescales and feeding behaviors, leading to refinements in the hypotheses regarding the potential roles of these neurons in feeding, food-seeking, valence coding and reinforcement learning, and suppression of competing drive states (Betley et al., 2015, Chen et al., 2015, Garfield et al., 2015, Palmiter, 2015, Seeley and Berridge, 2015) across multiple timescales and behavioral paradigms.

In summary, our recordings of spiking activity from identified classes of ARC neurons in awake mice provide direct support for previous hypotheses, based on in vitro recordings and in vivo manipulations, of slow, opposite changes in AgRP and POMC neuron spiking across hours, during slow changes in energy balance. In addition, our data suggest that AgRP and POMC neurons can be modulated on timescales inconsistent with a purely homeostatic role in feeding. In particular, the rapid drop in spiking activity of AgRP neurons at meal onset may reflect a termination of the drive to seek sources of food, while residual, persistent spiking in these neurons may reflect a sustained drive to consume food.

## Materials and methods

### Animals

All animal care and experimental procedures were approved by the Beth Israel Deaconess Medical Center Institutional Animal Care and Use Committee. We used 12 adult male mice, heterozygous for Cre recombinase under the control of the *Agrp* gene (*Agrp-Ires-cre;* Tong et al., 2008). Animals were housed at 22°C–24°C on a 12:12 light/dark cycle (light cycle: 6:00 am to 6:00 pm) with standard mouse chow and water provided ad libitum, unless specified otherwise.

### Surgery and viral injections

To selectively express channelrhodopsin-2 (ChR2) in AgRP neurons, we injected *Agrp-Ires-cre* mice with 200 nl of adeno-associated virus, serotype 9, carrying an inverted ChR2-mCherry flanked by double *loxP* sites (UPenn Vector Core, Philadelphia, PA) into the arcuate nucleus of the hypothalamus (ARC; coordinates relative to Bregma: anterior-posterior, −1.50 mm; dorsal-ventral, −5.80 mm; lateral, 0.25 mm). 3 weeks after viral injection, mice were prepared for awake, head-fixed electrophysiology recordings by surgical implantation of a head post and an optetrode microdrive (See ‘Optetrode electrophysiology’ section, below; see also Cohen et al., 2012) as follows: first, mice were anesthetized using isoflurane in 100% O_2_ (induction, 3%–5%; maintenance, 1%–2%) and placed into a stereotaxic apparatus (Kopf, Model 940 Small Animal Stereotaxic Instrument with Digital Display Console) on a heating pad (CWE). Ophthalmic ointment (Vetropolycin) was applied to the eyes. Using procedures identical to those described previously (Goldey et al., 2014), a two-pronged head post was affixed to the skull using C&B Metabond (Parkell; cat. no. 242-3200), and a 0.5-mm diameter burr hole was drilled over the mouse ARC. The optetrode was then implanted with distal electrode tips ending well above the arcuate nucleus (4.8 mm ventral to Bregma), and the implant was secured in place using a light-cured glue (FLOW-IT ALC part #N11VH, Pentron Clinical) around the craniotomy, followed by metabond and dental cement (Grip cement kit, powder and solvent; Dentsply; cat. no. 675570). Analgesia (0.5 mg/kg meloxicam, s.c) was administered post-operatively and on the following day.

### Post-surgery

Following recovery from surgery, mice were habituated to tolerate 1–2 hr of head restraint (typically requiring 3–4 days). Note that physiology studies in mice and primates commonly employ head-restraint, which enables more precise control and monitoring of feeding behavior and other behavioral and neurophysiological parameters (Paz et al., 2003, Niell and Stryker, 2010). It was previously shown that stress responses largely normalize after 3–4 days of habituation to restraint stress (Ma and Lightman, 1998). Furthermore, the fast changes in spiking activity we report cannot be explained by stress, since spontaneous activity over the course of tens of minutes (during experiments in Figure 2 in mice fed ad libitum, see below) was relatively stable under near-identical conditions.

### Free-feeding paradigm

A cohort of 9 mice, used to investigate diurnal rhythms of ARC neurons (Figure 2), had free access to food and maintained normal, *ad-libitum* feeding weight. In a typical session, recordings began with spontaneous activity for ∼5 min followed by a laser stimulation protocol (∼5–10 min). We then recorded spontaneous activity as long as recordings were stable. To minimize stress, head-fixation of habituated mice was restricted to <3 hr. Therefore, we restricted our daily recordings to either morning or afternoon, and advanced the electrodes between recordings to ensure that different neurons were recorded in each daily session.

### Instrumental feeding paradigm

A cohort of 5 mice, used to investigate feeding effects on ARC neurons (Figures 3–6), was maintained between 85 and 90% of *ad-libitum* weight (median across mice: 87.5%; averaged weight fluctuation within a mouse across sessions: 3.2 ± 1.8%). Mice were trained to consume a high-calorie liquid meal replacement (Ensure) from a lickspout, while head-fixed on a spherical treadmill. Licking was detected via disruption of an infrared beam positioned in front of the lickspout. Upon detection of a lick, a 10 µl drop of Ensure was released using a solenoid and MonkeyLogic software (Asaad and Eskandar, 2011) in Matlab. After ∼2–4 days of training, food-restricted mice would readily consume large quantities of Ensure. In a typical session, recordings began with spontaneous activity for ∼5 min followed by a laser photostimulation protocol (∼5–10 min). After a variable period of time (5–10 min), a lickspout was positioned in front of the mouse's snout. After an additional variable period of time (3–15 min), Ensure was made available, and the mouse subsequently engaged in instrumental licking for food reward (delivered in 10-µl increments). We then recorded activity as long as the mouse continued to eat (typically 1 hr, with consumption of 3–5 ml of Ensure, equivalent to 4.5–7.5 Cal). The hour-long recording typically includes ∼30 min of constant feeding, during which mice lick almost continuously and delivery of Ensure was contingent on detection of a lick. Of note, a minimal duration between Ensure drops was defined to be 2.5 s, even in periods of near-continuous licking behavior. This period of continuous feeding was then typically followed by more sparse bouts of feeding (Figure 6A, orange highlights). If the mouse stopped drinking for a substantial period of time, the software delivered a drop of Ensure to encourage additional licking. However, these Ensure drops constituted <15% of all drops delivered. When feeding further diminished, a second round of laser stimulation was performed to help with cell identification (Figure 5A). Mice were additionally given chow in their home cage (1.5–2 g, given between the hours of 6–8 PM) to help maintain a weight of approximately 87% of free-feeding weight. We confirmed that the chow was fully consumed by 8 AM the following morning, such that the subsequent recordings took place after at least 8 hr without access to food. In practice, since 2 g of chow is typically consumed within 4–5 hr, it is more likely that these recordings took place following at least 16 hr without feeding.

Note that we deliberately randomized the time until lickspout placement and the time from lickspout placement until the onset of Ensure availability, as well as occasionally interleaving feeding sessions with sessions of head-fixation without feeding, in order to avoid having the onset of lickspout placement and food delivery be perfectly predictable by prior cues (e.g., initial head restraint prior to recording), though it is likely that such cues nevertheless induced ‘predictive’ changes in firing.

### Optogenetic activation of AgRP neurons in head-fixed mice

A cohort of 5 mice was used to investigate whether optogenetic activation of AgRP neurons induced food consumption under head-fixed conditions (Figure 5). Mice were trained to consume Ensure from a lickspout, while head-fixed on a spherical treadmill. To this end, mice were first habituated for 1–2 days to head-fixation on the trackball. They were then mildly food restricted until their body weight decreased to 95% of their free-feeding weight. After another 1–2 days of habituating to feeding while head-fixed, mice were returned to their free-feeding weight. After at least 5 additional days, mice were tested. Each session began with spontaneous licking and consumption of up to 0.5 ml Ensure. After 30 min of baseline activity (during which mice rarely licked; Figure 5B), we photostimulated AgRP neurons for 30 min (20-ms pulses at 20 Hz; 1 s on / 3 s off), followed by an additional 30 min without photostimulation. During the session, licking and the delivery of Ensure were continuously recorded.

### Optetrode electrophysiology

We recorded extracellularly from multiple neurons simultaneously using a custom-built, 200-μm diameter optic fiber-coupled microdrive (an ‘optetrode’) with between four and eight manually constructed tetrodes (comprising of 4 twisted strands of electrode wire) attached to the sides of the fiber (Cohen et al., 2012). In the days following optetrode insertion (to depth of 4.8 mm ventral to Bregma), and prior to the beginning of recordings, tetrodes were gradually lowered by 0.5 mm (0.15 mm/day). All tetrodes were glued to the fiber with epoxy, such that the ends of the tetrodes were 400–600 μm beyond the end of the fiber. Each tetrode was then gold plated (Gold NC Solution, Neuralynx Inc), to reach a final impedance between 500 and 800 KΩ. Neural signals and time stamps for behavior were recorded using a Digital Lynx SX recording system (Neuralynx). Broadband signals from each wire, filtered between 0.1 and 9000  Hz, were recorded continuously at 32  kHz. To extract the timing of spikes, signals were band-pass filtered between 400 and 6000  Hz. Spikes were detected whenever a signal crossed a selected threshold value. For each electrode, the threshold was defined as four times an estimated noise level. The standard deviation of the background noise was estimated as the median of the absolute value of the band-pass filtered recording, divided by 0.6745 (Donoho and Johnstone, 1994, Quiroga et al., 2004). Spike waveforms for each electrode within a tetrode (a 1-ms epoch around each time stamp) were extracted using a broadband signal (300–9000  Hz) sampled at 32  kHz. This ensured that minimal information about spike waveform was lost to additional filtering.

Waveform spikes were then sorted offline using Neuralynx spike sorting software (SpikeSort) as follows: first, each spike waveform consisted of a 1-ms window surrounding its peak amplitude. Second, for each spike, we defined two features, amplitude of the peak and amplitude of the valley, for each of the four electrodes within a tetrode (a total of 8 features). Clusters were then defined according to these feature distributions, manually selecting the dimensions that best separated different clusters. We used several criteria to include a neuron in our data set. First, we inspected the ISI distribution. A histogram of the ISI distribution for the spikes within each cluster is expected to show a refractory period, that is, a dearth of spikes that occur within milliseconds of each other (Hill et al., 2011). Therefore, only clusters in which none of the ISIs were less than 1 ms and less than 5% of the ISIs were smaller than 5 ms were considered for further examination as candidates for single-units, thus ensuring minimal contamination. Second, the clustered waveforms were also inspected by eye to exclude those with aphysiological shapes. The waveform shape and amplitude were examined across the duration of the recording to ensure stability and reject the possibility of contamination by multiple neurons or potential loss of a neuron at an intermediate time within the recording. Finally, we performed cross-correlation between each spike waveform and the averaged waveform, and specified that the averaged correlation coefficient must exceed 0.95. To ensure stable recordings, we confirmed that the correlation coefficients between spikes in the first and last 5 min of recordings were not significantly different than those between the same number of randomly selected spikes across the recording. Recording sites were also verified histologically with electrolytic lesions at the termination of the experiment, when possible, using 15–20  s of 100 μA direct current, or by visualizing the optical fiber track (Figure 1A).

We adapted recent methods for optogenetic identification (Lima et al., 2009, Cohen et al., 2012, Kravitz et al., 2013) of well-isolated single-units, to classify these units into three categories, as follows. First, to classify AgRP neurons, we delivered blue-light photostimulation pulses at 20 Hz, a stimulation frequency shown to elicit feeding and as well as sustained spiking in ChR2-expressing AgRP neurons in vitro (Aponte et al., 2011). Specifically, we delivered 1-s-long trains of 20-ms light pulses at 20 Hz (wavelength: 473 nm; intensity: 5–20 mW/mm^2^), with 3 s between pulse trains (typically 50–100 trains were used at a given laser intensity). The laser beam was passed through a Pockels cell to ensure accurate control of laser pulse shape (<0.2 ms timing accuracy) and amplitude (calibrated with a power meter and a photodiode). To ensure that spontaneous and light-evoked waveforms originated from the same cell, we validated that the correlation coefficients of the cross-correlations between spontaneous and light-evoked waveforms were above 0.95 and were not significantly different than the correlation between pairs of spontaneous spike waveforms (see also Figure 3—figure supplement 2).

To determine whether a neuron showed a significant light-evoked response, we used a paired sample t-test comparing firing rates in the 2 s prior to a 20-Hz pulse train with the first half or the second half of the pulse train (p < 0.025, corrected for number of tests). This method was chosen because some clearly driven neurons showed more pronounced excitation after a delay of several 100's of milliseconds (see Figure 1—figure supplement 1; peri-stimulus time histograms show binned firing rates relative to laser train onset; estimated with 100-ms bins). For neurons that fired significantly below pre-train baseline (inhibited by AgRP neuron photostimulation) according to the above t-test, we added an additional criterion that the cells be suppressed by greater than 20% relative to baseline, which removed a subset of weakly but significantly inhibited cells (7% of all recorded cells). This class of cells was labeled ‘ARC_inh_’. Cells not significantly modulated according to the t-test were assigned to the ‘ARC_other_’ category. Finally, we noticed that in a small subset of recordings (<10%), the initial trial of a 1-s laser pulse train in the series of 1-s trains could lead to a sharp increase or decrease in firing that did not return to pre-photostimulation baseline until 0–2 min after the end of the final laser photostimulation trial. We reasoned that these effects were clearly laser-evoked (two-sample Kolmogorov–Smirnov (KS) test, p < 0.05), and thus, we also used this information in our classification. This additional criterion only changed the cell classification in 7% (2/33) of AgRP neurons and 12% (3/25) of ARC_inh_ neurons and did not affect the main conclusions of the study. While histology showed reasonably high penetrance of ChR2 expression in AgRP neurons, the ARC_other_ category may include a small subset of AgRP neurons lacking sufficient or any ChR2 expression, or that an insufficient intensity of light reached the tetrode on which the unit was recorded. Note that ARC_other_ and ARC_inh_ neurons were only included from recordings during or subsequent to identification of a putative AgRP neuron, to ensure that no neurons from regions dorsal to the ARC were included.

While many AgRP neurons showed classical entrainment to the pulse train at 20 Hz, some clearly laser-driven AgRP neurons did not show strong entrainment. Neurons with low excitability and low-spontaneous firing rates in vivo may be unsuitable for identification protocols demanding entrainment at high frequencies (Kravitz et al., 2013). This may be the case with AgRP neurons, whose excitability we found to be low in the morning (in ad libitum-fed mice, Figure 2) and following feeding (in food-restricted mice, Figure 3). Further, the intrinsic membrane properties of AgRP neurons have themselves been shown to be state dependent (Baver et al., 2014), thus providing an additional source of in vivo variability that may affect entrainment, but that is not present during most in vitro recordings (e.g., Aponte et al., 2011). Moreover, AgRP neurons might also not show entrainment due to other cell-intrinsic mechanisms that would only be influential in certain in vivo contexts, in the presence of strong, summating synaptic input (Jo et al., 2005). To quantify fast entrainment of spiking activity to each laser pulse, 50-ms cycle histograms were calculated in 5-ms bins (Figure 1—figure supplement 1C). To determine significant laser entrainment, a shuffling procedure was applied to spikes during individual laser cycles (20 ms of laser stimulation followed by 30 ms of no laser stimulation). We created a distribution of shuffled cycle histograms by shuffling the spikes within a given cycle 5000 times while maintaining the same total number of spikes per cycle. We compared the firing rate in our cycle histogram of individual 5-ms bins with the distribution of shuffled cycle histograms to determine if any bins were significantly modulated by photostimulation (p < 0.0001, corrected for number of neurons and number of bins).

### Data analysis

To identify whether ARC neurons were tonic firing, or had burst-like behavior involving occasional, short ISIs between longer ISIs, we used the Hartigan's dip test of unimodality on the distribution of the logarithm (to base 10) of each ISI (Figure 2—figure supplement 1; p < 0.05).

To quantify the changes in firing rate (estimated in 5-s bins) during the feeding paradigm, auROC timecourses (Cohen et al., 2012) were calculated for each cell (Figure 3D). This analysis compares the distribution of firing rates during a baseline period (up to 5 min prior to lickspout placement) with the distribution of firing rates post baseline, using a sliding window of 1 min. This analysis quantifies how discriminable these two distributions are. For example, if the two distributions of firing rates are completely non-overlapping, the auROC reflects an estimate of 1 (clear increase in firing; all post-baseline firing rate values are larger than all baseline firing rate values; red in Figure 3D) or 0 (clear decrease in firing; all post-baseline firing rate values are smaller than all baseline firing rate values; blue in Figure 3D), while an auROC estimate of 0.5 indicates that the distribution of baseline and post-baseline firing rates is indistinguishable (white in Figure 3D). This analysis effectively normalizes the responses across a population, allows concurrent visualization of neurons with very different firing rates, accounts for local firing rate variability, and speaks to the reliability of the difference between baseline and post-baseline time windows.

To quantify the percentage of AgRP, ARC_inh_, and ARC_other_ neurons modulated by placement of the lickspout or feeding, we compared the distribution of firing rates (estimated in 5-s bins) before lickspout placement (up to 5 min prior to lickspout placement) with the distribution of firing rates post-lickspout placement (‘food cue predictive responses’; up to 5 min post lickspout, but only including time bins prior to food availability; Figure 4) or post-Ensure delivery (early-feeding responses: 0–5 min following onset of Ensure availability; mid-feeding response: 5–15 min following onset of Ensure availability; late-feeding response: 15–45 min following onset of Ensure availability; Figure 3) via a two-sample KS-test (Figure 3E; p < 0.025).

For analyses of the relationship between firing rate and licks or lick bouts, we use multiple linear regression analysis. Simply stated, this analysis assumes a linear relationship between a neuron's firing and the occurrence of individual licks or lick bouts and estimates this relationship. The main advantage of this approach over the generation of lick-triggered (or bout-triggered) average firing rate histograms is that it assesses the impact of each lick or lick bout, irrespective of the occurrence of other prior or future licks or bouts. The analysis determines the linear ‘kernel’ (one for the relationship between firing and licking, another for the relationship between firing and lick bouts)—a set of coefficients that adjust the firing rate up or down at each moment in time relative to the occurrence of each individual lick or lick bout. If the relationship is indeed linear, then one should be able to perfectly predict the moment-by-moment firing of the neuron as the sum of the following three terms: (i) a fixed constant firing rate (in spikes/s), (ii) the convolution of the ‘lick’ kernel (units: spikes/s/lick; Figure 6C, left panels) with each individual lick, and (iii) the convolution of the ‘lick bout’ kernel (units: spikes/s/lick; Figure 6C, left panels) with each individual lick bout. Note that these kernels include coefficients at times both prior to and following onset of the lick or lick bout, to estimate changes in firing that precede and follow the licking event, respectively. The F-statistic assesses whether a given kernel coefficient at a given time relative to lick/bout onset explains a significant amount of variance. P-values were corrected for multiple comparisons (p < 0.05/19 = 0.0026, corrected for time bins, reflecting the 19 time bins at 2-Hz sampling rate, from −4.5 s to 4.5 s relative to lick or lick bout onset).

All statistical tests and analyses were performed using Matlab.

### Histology

At the conclusion of recordings, which lasted between 10 and 60 days, we performed histological verification of the recording site. In a subset of mice (5/12), an electrolytic lesion was made 400 µm above the final recording location by passing a mild current between two electrodes (25 mA for 30 s). Mice were given an overdose of tribromoethanol, perfused with 10% formalin, and brains were cut in 50-µm coronal sections. Sections were stained with 4′,6-diamidino-2-phenylindole (DAPI) to visualize nuclei. Recording sites, identified by the presence of the fiber tract and/or electrolytic lesion, were all verified to be among ChR2-mCherry-expressing AgRP neurons.

## References

[bib1] Aponte Y, Atasoy D, Sternson SM (2011). AGRP neurons are sufficient to orchestrate feeding behavior rapidly and without training. Nature Neuroscience.

[bib2] Arnauld E, du Pont J (1982). Vasopressin release and firing of supraoptic neurosecretory neurones during drinking in the dehydrated monkey. Pflugers Archiv.

[bib3] Arrigoni E, Saper CB (2014). What optogenetic stimulation is telling us (and failing to tell us) about fast neurotransmitters and neuromodulators in brain circuits for wake-sleep regulation. Current Opinion in Neurobiology.

[bib4] Asaad WF, Eskandar EN (2011). Encoding of both positive and negative reward prediction errors by neurons of the primate lateral prefrontal cortex and caudate nucleus. The Journal of Neuroscience.

[bib5] Atasoy D, Betley JN, Li WP, Su HH, Sertel SM, Scheffer LK, Simpson JH, Fetter RD, Sternson SM (2014). A genetically specified connectomics approach applied to long-range feeding regulatory circuits. Nature Neuroscienc.

[bib6] Atasoy D, Betley JN, Su HH, Sternson SM (2012). Deconstruction of a neural circuit for hunger. Nature.

[bib7] Bagnol D, Lu XY, Kaelin CB, Day HE, Ollmann M, Gantz I, Akil H, Barsh GS, Watson SJ (1999). Anatomy of an endogenous antagonist: relationship between Agouti-related protein and proopiomelanocortin in brain. The Journal of Neuroscience.

[bib8] Baver SB, Hope K, Guyot S, Bjorbaek C, Kaczorowski C, O'Connell KM (2014). Leptin modulates the intrinsic excitability of AgRP/NPY neurons in the arcuate nucleus of the hypothalamus. The Journal of Neuroscience.

[bib9] Becskei C, Lutz TA, Riediger T (2009). Diet-derived nutrients mediate the inhibition of hypothalamic NPY neurons in the arcuate nucleus of mice during refeeding. American Journal of Physiology. Regulatory, Integrative and Comparative Physiology.

[bib10] Berridge KC (2004). Motivation concepts in behavioral neuroscience. Physiology & Behavior.

[bib11] Betley JN, Cao ZF, Ritola KD, Sternson SM (2013). Parallel, redundant circuit organization for homeostatic control of feeding behavior. Cell.

[bib12] Betley JN, Xu S, Cao ZF, Gong R, Magnus CJ, Yu Y, Sternson SM (2015). Neurons for hunger and thirst transmit a negative-valence teaching signal. Nature.

[bib13] Bodosi B, Gardi J, Hajdu I, Szentirmai E, Obal F, Krueger JM (2004). Rhythms of ghrelin, leptin, and sleep in rats: effects of the normal diurnal cycle, restricted feeding, and sleep deprivation. American Journal of Physiology. Regulatory, Integrative and Comparative Physiology.

[bib14] Chen Y, Lin YC, Kuo TW, Knight Zachary A (2015). Sensory detection of food rapidly modulates arcuate feeding circuits. Cell.

[bib15] Cohen JY, Haesler S, Vong L, Lowell BB, Uchida N (2012). Neuron-type-specific signals for reward and punishment in the ventral tegmental area. Nature.

[bib16] Cone RD, Cowley MA, Butler AA, Fan W, Marks DL, Low MJ (2001). The arcuate nucleus as a conduit for diverse signals relevant to energy homeostasis. International Journal of Obesity and Related Metabolic Disorders.

[bib17] Cowley MA, Pronchuk N, Fan W, Dinulescu DM, Colmers WF, Cone RD (1999). Integration of NPY, AGRP, and melanocortin signals in the hypothalamic paraventricular nucleus: evidence of a cellular basis for the adipostat. Neuron.

[bib18] Cowley MA, Smart JL, Rubinstein M, Cerdan MG, Diano S, Horvath TL, Cone RD, Low MJ (2001). Leptin activates anorexigenic POMC neurons through a neural network in the arcuate nucleus. Nature.

[bib19] Craig W (1917). Appetites and aversions as constituents of instincts. Proceedings of the National Academy of Sciences of USA.

[bib20] Cummings DE, Purnell JQ, Frayo RS, Schmidova K, Wisse BE, Weigle DS (2001). A preprandial rise in plasma ghrelin levels suggests a role in meal initiation in humans. Diabetes.

[bib21] Davis JD (1989). The microstructure of ingestive behavior. Annals of the New York Academy of Sciences.

[bib22] de Araujo IE, Oliveira-Maia AJ, Sotnikova TD, Gainetdinov RR, Caron MG, Nicolelis MA, Simon SA (2008). Food reward in the absence of taste receptor signaling. Neuron.

[bib23] Dombeck DA, Khabbaz AN, Collman F, Adelman TL, Tank DW (2007). Imaging large-scale neural activity with cellular resolution in awake, mobile mice. Neuron.

[bib24] Donoho D, Johnstone J (1994). Ideal spatial adaptation by wavelet shrinkage. Biometrika.

[bib25] Ellis C, Moar KM, Logie TJ, Ross AW, Morgan PJ, Mercer JG (2008). Diurnal profiles of hypothalamic energy balance gene expression with photoperiod manipulation in the *Siberian hamster*, *Phodopus sungorus*. American Journal of Physiology Regulatory, Integrative and Comparative Physiology.

[bib26] Garfield AS, Li C, Madara JC, Shah BP, Webber E, Steger JS, Campbell JN, Gavrilova O, Lee CE, Olson DP, Elmquist JK, Tannous BA, Krashes MJ, Lowell BB (2015). A neural basis for melanocortin-4 receptor-regulated appetite. Nature Neuroscience.

[bib27] Goldey GJ, Roumis DK, Glickfeld LL, Kerlin AM, Reid RC, Bonin V, Schafer DP, Andermann ML (2014). Removable cranial windows for long-term imaging in awake mice. Nature Protocols.

[bib28] Gropp E, Shanabrough M, Borok E, Xu AW, Janoschek R, Buch T, Plum L, Balthasar N, Hampel B, Waisman A, Barsh GS, Horvath TL, Bruning JC (2005). Agouti-related peptide-expressing neurons are mandatory for feeding. Nature Neuroscience.

[bib29] Hill DN, Mehta SB, Kleinfeld D (2011). Quality metrics to accompany spike sorting of extracellular signals. The Journal of Neuroscience.

[bib30] Histed MH, Carvalho LA, Maunsell JH (2012). Psychophysical measurement of contrast sensitivity in the behaving mouse. Journal of Neurophysiology.

[bib31] Jo YH, Chen YJ, Chua SC, Talmage DA, Role LW (2005). Integration of endocannabinoid and leptin signaling in an appetite-related neural circuit. Neuron.

[bib32] Kentish SJ, Frisby CL, Kennaway DJ, Wittert GA, Page AJ (2013). Circadian variation in gastric vagal afferent mechanosensitivity. The Journal of Neuroscience.

[bib33] Kentros CG, Agnihotri NT, Streater S, Hawkins RD, Kandel ER (2004). Increased attention to spatial context increases both place field stability and spatial memory. Neuron.

[bib34] Kim JD, Leyva S, Diano S (2014). Hormonal regulation of the hypothalamic melanocortin system. Frontiers in Physiology.

[bib35] Krashes MJ, Koda S, Ye C, Rogan SC, Adams AC, Cusher DS, Maratos-Flier E, Roth BL, Lowell BB (2011). Rapid, reversible activation of AgRP neurons drives feeding behavior in mice. The Journal of Clinical Investigation.

[bib36] Krashes MJ, Shah BP, Koda S, Lowell BB (2013). Rapid versus delayed stimulation of feeding by the endogenously released AgRP neuron mediators GABA, NPY, and AgRP. Cell Metabolism.

[bib37] Krashes MJ, Shah BP, Madara JC, Olson DP, Strochlic DE, Grafield AS, Vong L, Pei H, Watabe-Uchida M, Uchida N, Liberles SD, Lowell BB (2014). An excitatory paraventricular nucleus to AgRP neuron circuit that drives hunger. Nature.

[bib38] Kravitz AV, Owen SF, Kreitzer AC (2013). Optogenetic identification of striatal projection neuron subtypes during in vivo recordings. Brain Research.

[bib39] Lima SQ, Hromadka T, Znamenskiy P, Zador AM (2009). PINP: a new method of tagging neuronal populations for identification during in vivo electrophysiological recording. PLOS ONE.

[bib40] Liu T, Kong D, Shah Bhavik P, Ye C, Koda S, Saunders A, Ding Jun B, Yang Z, Sabatini Bernardo L, Lowell Bradford B (2012). Fasting activation of AgRP neurons requires NMDA receptors and involves spinogenesis and increased excitatory tone. Neuron.

[bib41] Lu XY, Shieh KR, Kabbaj M, Barsh GS, Akil H, Watson SJ (2002). Diurnal rhythm of agouti-related protein and its relation to corticosterone and food intake. Endocrinology.

[bib42] Luquet S, Perez FA, Hnasko TS, Palmiter RD (2005). NPY/AgRP neurons are essential for feeding in adult mice but can be ablated in neonates. Science.

[bib43] Ma XM, Lightman SL (1998). The arginine vasopressin and corticotrophin-releasing hormone gene transcription responses to varied frequencies of repeated stress in rats. The Journal of Physiology.

[bib44] Mistlberger RE (2009). Food-anticipatory circadian rhythms: concepts and methods. The European Journal of Neuroscience.

[bib45] Moran TH, Tamashiro KL (2007). Curt Richter: spontaneous activity and food intake. Appetite.

[bib46] Morton GJ, Schwartz MW (2001). The NPY/AgRP neuron and energy homeostasis. International Journal of Obesity and Related Metabolic Disorders.

[bib47] Niell CM, Stryker MP (2010). Modulation of visual responses by behavioral state in mouse visual cortex. Neuron.

[bib48] Palmiter R (2015). Hunger logic. Nature Neuroscience.

[bib49] Paz R, Boraud T, Natan C, Bergman H, Vaadia E (2003). Preparatory activity in motor cortex reflects learning of local visuomotor skills. Nature Neuroscience.

[bib50] Quiroga RQ, Nadasdy Z, Ben-Shaul Y (2004). Unsupervised spike detection and sorting with wavelets and superparamagnetic clustering. Neural Computation.

[bib51] Saper CB, Chou TC, Elmquist JK (2002). The need to feed: homeostatic and hedonic control of eating. Neuron.

[bib52] Schone C, Apergis-Schoute J, Sakurai T, Adamantidis A, Burdakov D (2014). Coreleased orexin and glutamate evoke nonredundant spike outputs and computations in histamine neurons. Cell Reports.

[bib53] Seeley RJ, Berridge KC (2015). The hunger games. Cell.

[bib54] Siegle JH, Wilson MA (2014). Enhancement of encoding and retrieval functions through theta phase-specific manipulation of hippocampus. eLife.

[bib55] Stricker EM, Hoffmann ML (2007). Presystemic signals in the control of thirst, salt appetite, and vasopressin secretion. Physiology & Behavior.

[bib56] Summerlee AJ, Lincoln DW (1981). Electrophysiological recordings from oxytocinergic neurones during suckling in the unanaesthetized lactating rat. The Journal of Endocrinology.

[bib57] Tan K, Knight ZA, Friedman JM (2014). Ablation of AgRP neurons impairs adaption to restricted feeding. Molecular Metabolism.

[bib58] Thorn CA, Graybiel AM (2014). Differential entrainment and learning-related dynamics of spike and local field potential activity in the sensorimotor and associative striatum. The Journal of Neuroscience.

[bib59] Tong Q, Ye CP, Jones JE, Elmquist JK, Lowell BB (2008). Synaptic release of GABA by AgRP neurons is required for normal regulation of energy balance. Nature Neuroscience.

[bib60] Tschop M, Smiley DL, Heiman ML (2000). Ghrelin induces adiposity in rodents. Nature.

[bib61] van den Top M, Lee K, Whyment AD, Blanks AM, Spanswick D (2004). Orexigen-sensitive NPY/AgRP pacemaker neurons in the hypothalamic arcuate nucleus. Nature Neuroscience.

[bib62] Varela L, Horvath TL (2012). Leptin and insulin pathways in POMC and AgRP neurons that modulate energy balance and glucose homeostasis. EMBO Reports.

[bib63] Wang L, Saint-Pierre DH, Tache Y (2002). Peripheral ghrelin selectively increases Fos expression in neuropeptide Y - synthesizing neurons in mouse hypothalamic arcuate nucleus. Neuroscience Letters.

[bib64] Wang Q, Liu C, Uchida A, Chuang JC, Walker A, Liu T, Osborne-Lawrence S, Mason BL, Mosher C, Berglund ED, Elmquist JK, Zigman JM (2014). Arcuate AgRP neurons mediate orexigenic and glucoregulatory actions of ghrelin. Molecular Metabolism.

[bib65] Willesen MG, Kristensen P, Rømer J (1999). Co-localization of growth hormone secretagogue receptor and NPY mRNA in the arcuate nucleus of the rat. Neuroendocrinology.

[bib66] Yang Y, Atasoy D, Su HH, Sternson SM (2011). Hunger states switch a flip-flop memory circuit via a synaptic AMPK-dependent positive feedback loop. Cell.

[bib67] Yaswen L, Diehl N, Brennan MB, Hochgeschwender U (1999). Obesity in the mouse model of pro-opiomelanocortin deficiency responds to peripheral melanocortin. Nature Medicine.

[bib68] Zhan C, Zhou J, Feng Q, Zhang JE, Lin S, Bao J, Wu P, Luo M (2013). Acute and long-term suppression of feeding behavior by POMC neurons in the brainstem and hypothalamus, respectively. The Journal of Neuroscience.

[bib69] Zigman JM, Elmquist JK (2003). Minireview: from anorexia to obesity—the yin and yang of body weight control. Endocrinology.

